# A Bioinspired Three‐Dimensional High‐Curvature Nano‐Interface Integrated Microfluidic Chip for Small Extracellular Vesicles Enrichment and Machine Learning‐Assisted Prostate Cancer Precision Diagnosis

**DOI:** 10.1002/advs.202521178

**Published:** 2026-05-14

**Authors:** Le Wang, Yizhong Liang, Manan Sulaiman, Jiaqi Du, Ming Jiang, Zhihua Wang, Xu Yu, Li Xu

**Affiliations:** ^1^ Department of Pharmacy Tongji Hospital Tongji Medical College Huazhong University of Science and Technology Wuhan China; ^2^ Tongji School of Pharmacy Huazhong University of Science and Technology Wuhan China; ^3^ Department of Urology Tongji Hospital Tongji Medical College Huazhong University of Science and Technology Wuhan China; ^4^ Taikang Tongji (Wuhan) Hospital Wuhan China

**Keywords:** affinity interaction, machine learning, prostate cancer diagnosis, size recognition, small extracellular vesicles

## Abstract

The efficient and unbiased isolation of small extracellular vesicles (sEVs) from complex biological fluids remains a major obstacle for clinical diagnostics. Here, we report a bioinspired microfluidic chip that integrates a three‐dimensional high‐curvature TiO_2_ nano‐interface (3D Hic‐TiO_2_) with a biotin‐modified artificial insertion peptide (BAIP) modification for rapid enrichment of sEV. Bowl‐shaped TiO_2_ nanospheres fabricated via electrospray provide topological nanotraps that match the size and curvature of sEVs, enabling efficient size‐selective capture. Coupled with BAIP‐mediated membrane affinity and herringbone‐induced chaotic mixing, the BAIP‐TiO_2_‐Chip achieved >90% capture efficiency within 5 min. Redox‐responsive BAIP variants enabled mild release of intact sEVs for downstream analysis. When the BAIP‐TiO_2_‐Chip was applied to plasma samples from clinical prostate cancer (PCa) patients, mass spectrometry‐based proteomic profiling revealed 110 differentially expressed sEV‐associated proteins, including candidates involved in immune regulation and cell adhesion. In parallel, simultaneous quantification of *PSA* and *PSMA* mRNAs in sEVs could be achieved. Assisted by machine learning, a boosted decision tree model achieved 80% diagnostic accuracy in distinguishing PCa from benign conditions and healthy donors. This work presents a versatile platform for sEV isolation, enabling both transcriptomic mRNA analysis and proteomic profiling, and provides new molecular insights into PCa for improved early diagnosis.

## Graphical abstract

A biotin‐modified artificial insertion peptide functionalized three‐dimensional high‐curvature‐TiO_2_ nano‐interface was engineered in a microfluidic chip to improve the isolation efficiency of small extracellular vesicles (sEVs). This chip balanced affinity, releasability, and extendibility, enabling high‐throughput recovery of sEVs for downstream applications including protein profiling and mRNA analysis.

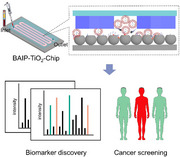

## 1 Introduction

Efficient and selective separation of target analytes from complex matrices remains a kernel challenge for a successful analytical procedure, where the rational design of advanced interfaces is crucial for enhancing capture efficiency, specificity, and detection reliability [1, 2, 3]. An ideal high‐performance isolation interface should not only provide strong and specific affinity interactions but also enable effective minimizing nonspecific adsorption [4, 5, 6]. Meanwhile, it should allow for reversible binding to facilitate the controlled release and recovery of target analytes. Despite considerable progress, current interface engineering strategies often struggle to fulfill all these criteria simultaneously. Approaches, such as microfluidic‐assisted mass transfer [7, 8, 9], multivalent ligand assembly [10, 11, 12, 13], and stimuli‐responsive release systems [14, 15, 16], have been investigated to address this challenge. However, these methods typically focus on individual performance parameters, such as enhancing transport, improving binding strength, or enabling controlled release, but fall short of delivering a comprehensive solution that integrates affinity, selectivity, and reversibility within a single interface platform.

To address these issues, a promising strategy lying in the construction of bioinspired three‐dimensional high‐curvature micro‐ or nano‐interfaces that mimic the morphology and size of the target species was proposed. By creating interfaces that match the structural features of the target analyte, such system can enhance the precision of recognition events, significantly improving sensitivity and selectivity. Such shape‐ and size‐complementary designs leverage a “lock‐and‐key” binding principle, wherein the spatial compatibility between the interface (e.g., pores, grooves, or protrusions) and the target facilitates physical confinement and recognition [2, 17]. Although these engineered interfaces provide structural advantages for selective binding, their performance can be further elevated through integration with microfluidic platforms [6]. Microfluidic chips provide precise control over fluid dynamics, enabling enhanced interactions between the analyte and the functionalized interface [18, 19]. This integration creates a synergistic system in which the confined flow environment amplifies the geometric and chemical recognition capabilities of the bioinspired interface, resulting in a performance that exceeds the additive effects of the individual components. The microscale flow conditions within microfluidic channels increase the collision frequency between the target analytes and the structured interface, which not only improves capture efficiency, especially for rare targets, but also reduces the required sample volume and processing time [20, 21].

Small extracellular vesicles (sEVs), including exosomes and microvesicles, are nanoscale lipid bilayer vesicles secreted by a variety of cell types and involved in intercellular communication, immune regulation, and pathological processes such as tumor progression and metastasis [22, 23, 24]. Due to their enrichment of disease‐specific biomolecules, like proteins, lipids, and RNAs, sEVs have attracted growing interest as promising biomarkers for early diagnosis, prognosis evaluation, and therapeutic monitoring [25, 26, 27]. However, their effective isolation and analysis from complex biological matrices, such as plasma or urine, remain challenging [28, 29]. To achieve high performance in sEV capture, the interface designs should prioritize structural compatibility with sEVs, particularly in terms of size and curvature. Designing structurally compatible interfaces, especially with matched size and curvature, may substantially improve sEV capture without relying solely on biochemical affinity. In addition, incorporating these interfaces into microfluidic systems may further enhance selectivity and throughput, while reducing nonspecific interactions [6, 30, 31, 32, 33, 34]. Several studies have demonstrated the feasibility of this strategy for targeting sEVs. For instance, Takeuchi et al. reported a molecular imprinting‐based dynamic molding method to construct nanocavities capable of size‐selective recognition of sEVs [35, 36]. Yang et al. fabricated magnetic colloid antibody systems via co‐polymerization of organosilane monomers and sEV templates, allowing for size‐specific enrichment of these biological nanoparticles [37]. Nevertheless, the complexity of sEVs or nanosphere self‐assembly, the multi‐step imprinting process, and difficulties in template removal limit their scalability and practical application to a large degree.

**SCHEME 1:**
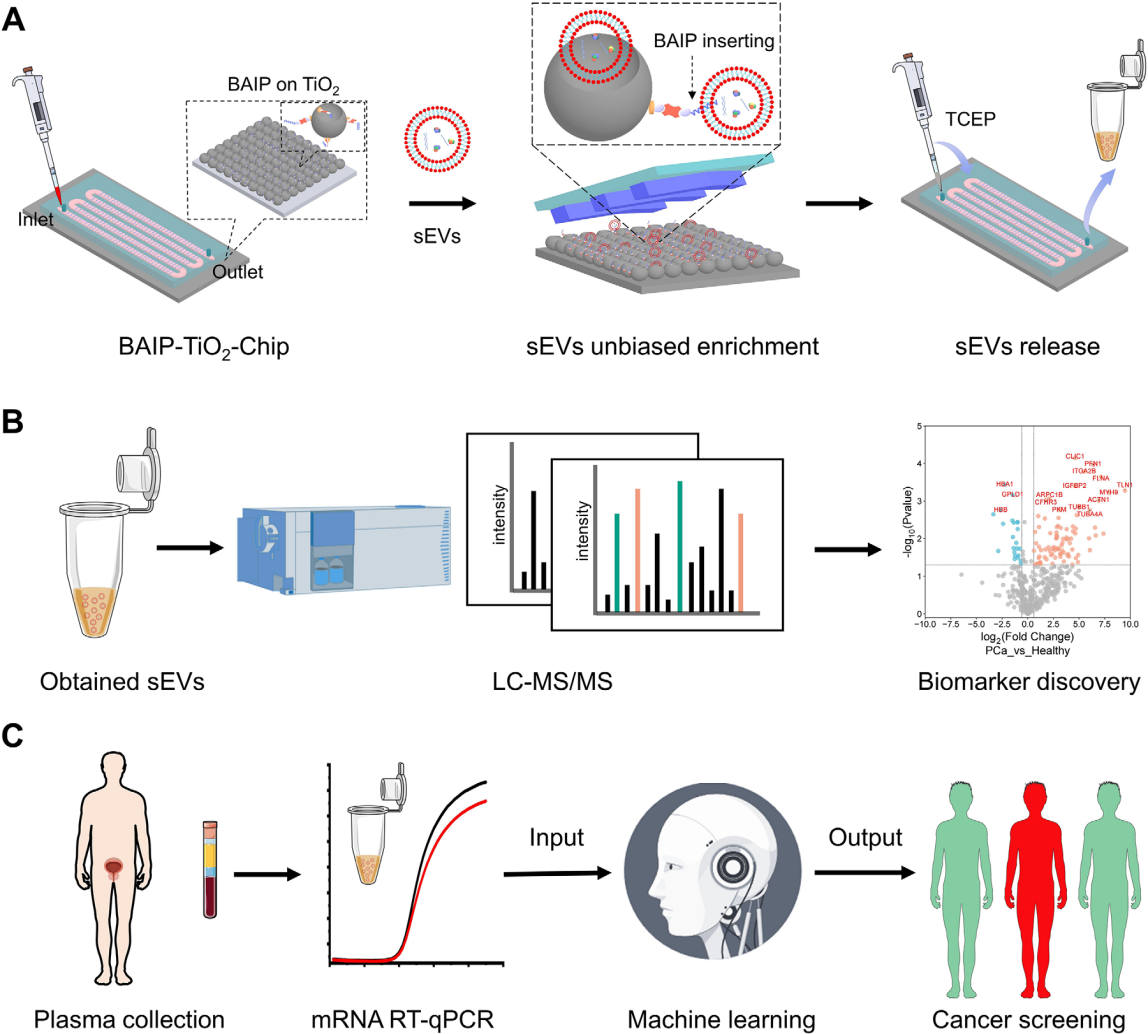
(A) Schematic illustration of the BAIP‐TiO_2_‐Chip for isolation and release of sEVs. (B) Downstream proteomic profiling of PCa sEVs. (C) mRNA analysis of sEVs with the assistance of machine learning for PCa precision diagnosis.

Herein, in this study, we designed and prepared a bioinspired **t** hree‐**d** imensional **hi** gh‐**c** urvature‐**TiO _2_** nano‐interface (3D Hic‐TiO_2_ interface) which was integrated into a microfluidic chip (TiO_2_‐Chip) for the isolation and enrichment of sEVs. The 3D bowl‐shaped TiO_2_ nanosphere interface was readily prepared on a large scale through electrospinning technology, offering a high density of binding sites and a topologically compatible surface for recognizing the size and curvature of sEVs. We further functionalized the interface using our previously developed **b** iotin‐modified **a** rtificial **i** nsertion **p** eptide (BAIP) [38] in order to enhance the specificity and binding affinity of the interface toward the sEVs. Compared with antibody‐antigen‐based capture approaches, the BAIP insertion strategy showed reduced dependence on antigen expression abundance and reduced susceptibility to epitope masking, which was advantageous for heterogeneous sEV populations. The resulting microfluidic chip (BAIP‐TiO_2_‐Chip) enabled rapid, unbiased, and highly efficient capture of sEVs, as illustrated in Scheme 1. This performance arose from the synergistic effects of three key mechanisms: (1) shape and size complementarity between the sEVs and the nanostructured interface, (2) membrane affinity recognition through BAIP modification, and (3) enhanced collision frequency facilitated by the herringbone‐induced chaotic mixing within the microfluidic channel. Importantly, the unbiased capture capability allowed for the retention of diverse sEV subpopulations, minimizing the loss of those with distinct molecular profiles, a common limitation from sEV heterogeneity [39, 40, 41, 42]. The platform also supported high‐throughput isolation and efficient recovery of sEVs, making it suitable for downstream applications including functional studies and multiple analysis, like mass spectrometry‐based proteomic analysis and qPCR based messenger RNAs (mRNAs) analysis, etc. These capabilities facilitated the discovery of sEV function and novel biomarkers for sEV‐based cancer diagnostics. Furthermore, we applied the BAIP‐TiO_2_‐Chip to isolate sEVs from the plasma of prostate cancer (PCa) patients, enabling the simultaneous detection of multiple mRNAs contained within the vesicles. Assisted by machine learning, we trained a boosted decision tree model using four selected features, which improved the diagnostic accuracy for PCa, achieving an overall accuracy of 80% on the test set. This work provides a proof‐of‐concept strategy for constructing bioinspired interfaces tailored to the physicochemical features of target analytes. Such an approach holds a broad potential for developing efficient and specific isolation platforms for a wide range of biomedical applications.

## 2 Results and Discussion

### 2.1 Characterization of Bioinspired 3D Hic‐TiO_2_ Interface Integrated Microfluidic Chip (TiO_2_‐Chip)

**FIGURE 1:**
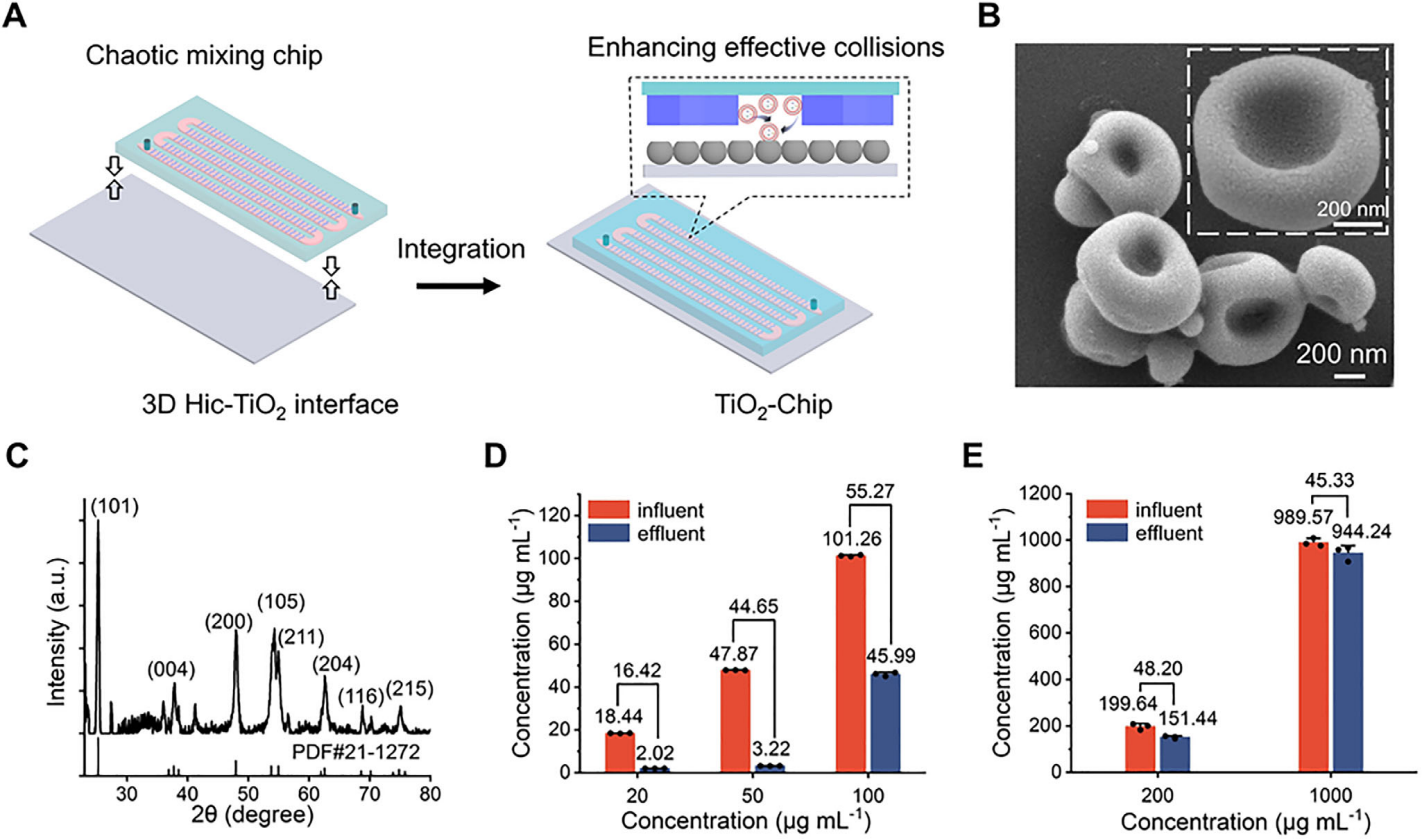
Characterization of BAIP‐TiO_2_‐Chip. (A) The fabrication scheme of TiO_2_‐Chip. (B) SEM image (inset: the high magnification) of the 3D Hic‐TiO_2_ interface. (C) XRD pattern of the 3D Hic‐TiO_2_. (D) The concentration of SA before and after incubation with BSA‐biotin on TiO_2_‐Chip. (E) The concentration of BAIP before and after incubation with SA on BAIP‐TiO_2_‐Chip.

To enhance sEVs capture efficiency, we aimed to construct a high‐curvature nano‐interface which matched the size and morphology of sEVs and integrate it into microfluidic chip. Since sEVs were typically in size from 30 to 200 nm with spherical morphologies, we proposed designing bioinspired interfaces that align with the size and shape of sEVs, creating nanotraps with dimensions close to sEVs. This design was intended to enhance the capture efficiency of sEVs through a 3D size recognition effect, as the interface provided more 3D contact sites and size‐complementary spaces for sEVs capture. Serendipitously, we found that a micro‐interface made of TiO_2_ nanospheres (TiO_2_ interface) could be efficiently produced on a large scale using a cost‐effective electrospray method (Figure S1). This formation process was observed during electrospray deposition of the TiO_2_ precursor solution. During the formation of bowl‐shaped TiO_2_, the solution that exited the nozzle broke into discrete droplets that were propelled toward a positively charged collector plate. As they traveled through the air, the solvent on the front surface of each droplet evaporated more rapidly due to airflow‐induced effects. This uneven evaporation led to deformation of the charged droplets, gradually reshaping them from an initial spherical form into a characteristic bowl‐shaped structure [43]. As displayed, the 3D Hic‐TiO_2_ interface formed by these TiO_2_ nanospheres on glasses exhibited uniform roughness, as observed under an optical microscope (Figure S2). Additionally, the nanointerface integrated microfluidic chips (TiO_2_‐Chip) were translucent, making them suitable for fluorescence imaging (Figure 1A; Figure S2). The TiO_2_‐interface displayed a single‐sided nanoconcave structure, with the size of nanocavities (∼200 nm) comparable to that of sEVs (Figure 1B). These nanocavities offered additional 3D contact sites and size‐complementary nanotraps for sEVs, facilitating the topological interactions between the substrate and sEVs.

The transmission electron microscopic (TEM) elemental mapping images revealed a uniform distribution of Ti and O elements across the 3D Hic‐TiO_2_ (Figure S3). The X‐ray diffraction (XRD) pattern of the 3D Hic‐TiO_2_ was consistent with the standard anatase TiO_2_ (PDF#21‐1272), with no impurity peaks observed (Figure 1C), confirming the high purity of the synthesized nanospheres. To determine the elemental composition and chemical state of the 3D Hic‐TiO_2_, X‐ray photoelectron spectroscopic (XPS) measurement was conducted (Figure S4). The survey spectrum exhibited four predominant peaks at 284.0 eV (C 1s), 457.8 eV (Ti 2p), 529.1 eV (O 1s), and 496.1 eV (Na Auger peak). High‐resolution spectra revealed that the binding energy peaks of Ti 2p_3/2_ and Ti 2p_1/2_ appeared at 457.8 eV and 463.4 eV, respectively. The O 1s spectrum displayed two characteristic peaks at 529.0 and 531.0 eV, corresponding to O in the TiO_2_ lattice and ‐OH groups on the TiO_2_ surface, respectively.

To functionalize the 3D Hic‐TiO_2_ interface on the microfluidic chip, BSA‐biotin was initially adsorbed through the nonspecific interactions according to the previous literature (Figures S5 and S6) [44]. Subsequently, BAIP was anchored onto the TiO_2_‐Chip through affinity interaction between biotin and streptavidin, resulting in the formation of the BAIP‐TiO_2_‐Chip. We optimized the SA modification (Figure S7A), where a concentration of 20 µg mL^−1^ showed insufficient coverage, while 50 µg mL^−1^ ensured full coverage of the entire microchannel. Further increasing the SA concentration to 100 µg mL^−1^ resulted in minimal difference compared to 50 µg mL^−1^ modification (Figure 1D; Figure S7B,C). Next, the maximum modification amount of BAIP was evaluated using high concentrations of BAIP (200 µg mL^−1^ and 1 mg mL^−1^). After modification, the BAIP concentrations in the solution decreased 48.2 and 45.3 µg mL^−1^, respectively (Figure 1E; Figure S8), demonstrating that BAIP was almost saturated at these two concentrations. The BAIP density on the 3D Hic‐TiO_2_ interface was calculated to be 6.9 × 10^13^ molecules cm^−2^, exhibited comparable or super‐modification density compared to other reported methods for peptide modification [45, 46], confirming its high‐density functionalization on the microfluidic chip.

### 2.2 Rapid and Efficient Capture of sEVs on BAIP‐TiO_2_‐Chip

**FIGURE 2:**
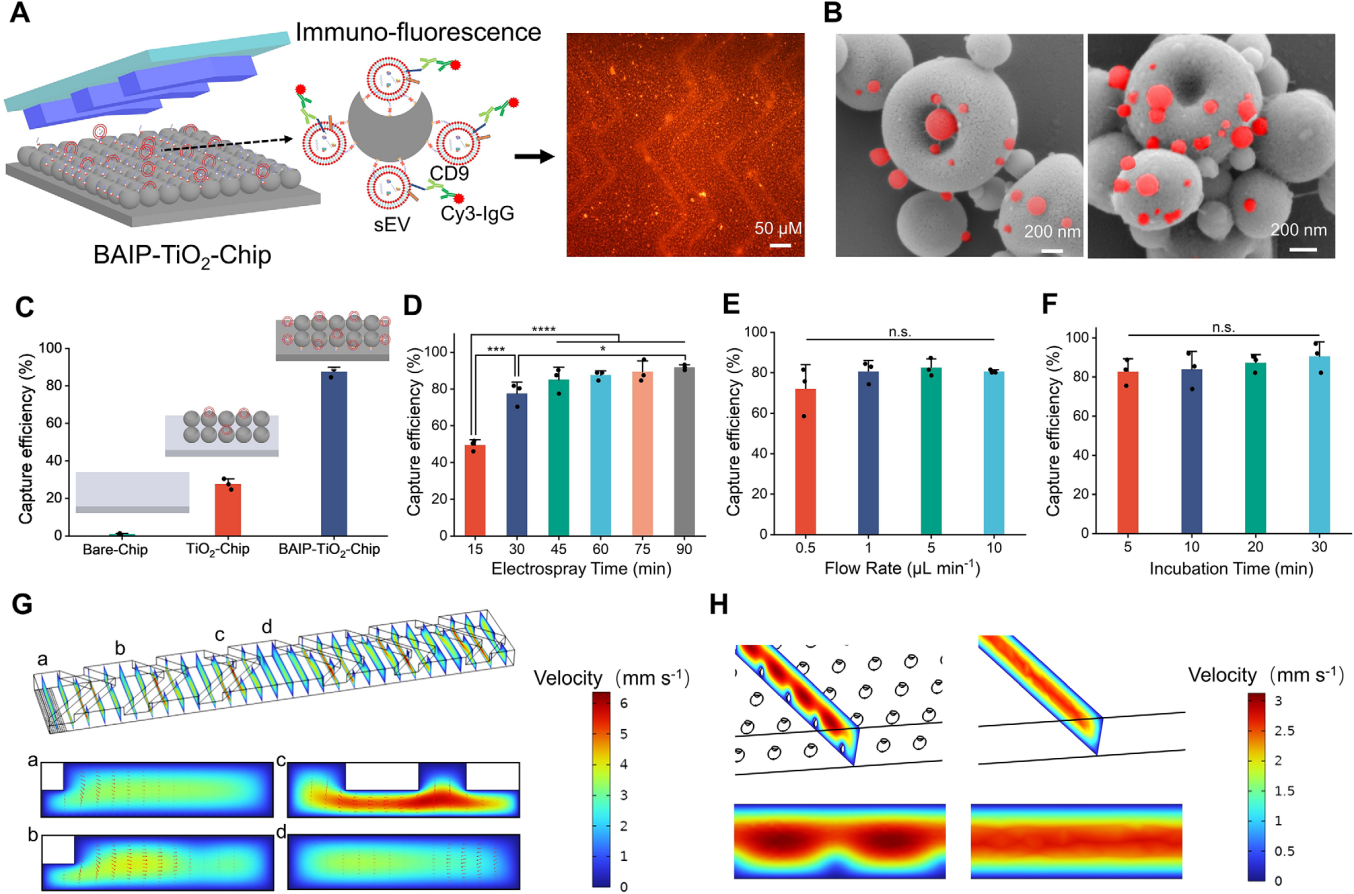
The isolation of sEVs. (A) Diagram of immuno‐fluorescence characterization of sEVs captured on BAIP‐TiO_2_‐Chip and the fluorescence image for MCF‐7 sEVs on BAIP‐TiO_2_‐Chip with CD9 and Cy3‐labeled goat anti‐mouse IgG (Cy3‐IgG) antibodies. (B) SEM images of sEVs captured on BAIP‐TiO_2_‐Chip. (C) Comparison of sEVs capture efficiency between Bare‐Chip, TiO_2_‐Chip, and BAIP‐TiO_2_‐Chip. (D) The capture efficiency of BAIP‐TiO_2_‐Chip with different electrospray time of 3D Hic‐TiO_2_ toward MCF‐7 sEVs. (E) The capture efficiency of BAIP‐TiO_2_‐Chip toward MCF‐7 sEVs at different flow rates. (F) The capture efficiency of BAIP‐TiO_2_‐Chip toward MCF‐7 sEVs with different incubation time. (G) Overall flow velocity distribution across the chip and corresponding magnified view of a specific region. (H) Comparative analysis of localized flow velocity distribution with and without 3D Hic‐TiO_2_, presented in magnified detail.

Except for the construction of the 3D Hic‐TiO_2_ interface for topological size recognition and BAIP‐based affinity recognition, we also adopted a herringbone microfluidic chip to enhance the collision between sEVs and the functionalized interface, further maximizing sEVs capture efficiency (Figure S9). The breast cancer MCF‐7 cells derived sEVs were used as model sEVs to evaluate the capture performance of our BAIP‐TiO_2_‐Chip (Figure S10). Initially, immuno‐fluorescence and SEM methods were used to assess the feasibility of BAIP‐TiO_2_‐Chip for the rapid capture of sEVs. The immuno‐fluorescence method was applied to detect the tetraspanin proteins, CD9, on sEVs (Figure 2A). A significant difference in fluorescence intensity of CD9 between the BAIP‐TiO_2_‐Chip and the control group was observed, indicating that the BAIP‐TiO_2_‐Chip effectively captured sEVs (Figure S11). Additionally, as shown in the SEM images, a substantial number of MCF‐7 sEVs were present on the BAIP‐TiO_2_‐Chip (Figure 2B; Figure S12), confirming its ability to capture sEVs.

To further elucidate the synergistic effect between the topological size recognition provided by 3D Hic‐TiO_2_ interface and the affinity‐based recognition conferred by BAIP toward sEVs, we systematically compared the capture efficiencies of sEVs using three types of microfluidic chips, including a bare glass‐based microfluidic chip (Bare‐Chip), a 3D Hic‐TiO_2_ interface integrated microfluidic chip without BAIP modification (TiO_2_‐Chip) and the fully functionalized BAIP‐TiO_2_‐Chip (Figure 2C). As anticipated, the Bare‐Chip showed negligible sEVs capture (0.91% ± 0.46%), due to the absence of any specific recognition elements. However, the TiO_2_‐Chip still captured 27.57% ± 0.46% of sEVs, despite that the 3D Hic‐TiO_2_ interface was passive with BSA, which effectively ruled out binding between TiO_2_ and sEVs phospholipid membranes. This result suggested that topological size recognition between the bowl‐shaped structures and sEVs contributed significantly to their capture. Notably, the BAIP‐TiO_2_‐Chip exhibited a markedly enhanced capture efficiency of 87.45% ± 2.47%, which could be attributed to the synergistic effect among size‐dependent topological recognition, BAIP‐mediated affinity interactions, and the enhanced collision frequency resulting from chaotic mixing induced by the herringbone microstructures within the microfluidic chip (Figure 2C) [8, 14, 18, 47]. Collectively, the 3D Hic‐TiO_2_ interface not only offered an enlarged surface area for BAIP immobilization but also featured concave bowl‐like architectures that closely matched the size of sEVs, enabling effective size‐based recognition and contributing to the overall enhancement in capture performance.

Next, to guarantee the optimal sEVs capture, the electrospray time for creating 3D Hic‐TiO_2_ interface, and the influence factors for sEVs capture including the flow rate and the incubation time were optimized. As shown in Figure 2D, the sEVs capture efficiency increased from 49.43% ± 2.97% to 91.73% ± 1.43% as the electrospray time increased from 15 to 90 min, reaching a plateau at 45 min. The reason might be attributed to the high density of 3D Hic‐TiO_2_ spheres obtained with the extending electrospray time. The capture efficiency was 71.98% ± 11.99%, 80.61% ± 5.45%, 82.53% ± 4.40%, and 80.61% ± 0.83% at flow rates of 0.1, 1, 5, and 10 µL min^−1^, respectively (Figure 2E). Only minimal variation on the capture efficiency was observed with the varied flow rates on the microfluidic chip, indicating that the BAIP‐TiO_2_‐Chip could withstand high shear forces at elevated flow rates while maintaining effective capture performance, suggesting that our chip could be applied for the quick capture of sEVs, which might be due to the high affinity between BAIP and sEVs. Additionally, the impact of incubation time on capture efficiency of the BAIP‐TiO_2_‐Chip was examined. As shown in Figure 2F, the capture efficiency increased from 82.69% ± 4.40% to 90.41% ± 7.58% when the incubation time was extended from 5 to 30 min. However, no significant improvement in capture efficiency was observed beyond 5 min of incubation, indicating that the BAIP‐TiO_2_‐Chip was capable of rapidly capturing sEVs. In contrast, the gold standard method of ultracentrifugation achieved a capture efficiency of only 14.83% ± 5.47% and required approximately 4 h of processing time, indicating that the BAIP‐TiO_2_‐Chip markedly reduced the isolation time while substantially improving sEVs capture efficiency. Notably, in our previous study [38], about 76% of sEVs were captured within 20 min by using BAIP modified polystyrene beads (BAIP‐PS). In contrast, the BAIP‐TiO_2_‐Chip achieved higher capture efficiency (∼90%) in only 5 min. This improvement was primarily attributed to the increased frequency of collisions between the sEVs and 3D Hic‐TiO_2_ interface functionalized with BAIP inside the microfluidic chip. Meanwhile, in the fluid simulation, the fluid velocity near the herringbone protrusions was higher than in regions without such structures, confirming that the herringbone design enhanced fluid mixing and promoted efficient collisions between the target and the substrate (Figure 2G). The 3D Hic‐TiO_2_ structures reduced the local fluid velocity in their vicinity (Figure 2H), thereby prolonging the interaction time between sEVs and the BAIP functionalized substrate. Furthermore, the size‐dependent topological recognition further promoted selective and efficient binding of sEVs. Expectedly, the combination of these features would significantly reduce the required incubation time while simultaneously improve the overall capture performance.

To validate the unbiased capture of sEVs by the BAIP‐TiO_2_‐Chip, its isolation performance was compared with that of an EpCAM antibody modified TiO_2_‐Chip using sEVs derived from three breast cell lines with distinct EpCAM expression levels, including MCF‐7, MDA‐MB‐231, and MCF‐10A cells. The BAIP‐TiO_2_‐Chip achieved consistently high isolation efficiencies (90.37% ± 1.87%, 89.57% ± 2.42%, and 82.15% ± 1.91%, respectively), and the proportion of EpCAM‐positive sEVs remained essentially unchanged after isolation, showing minimal dependence on EpCAM expression (Figures S13 and S14). In contrast, the EpCAM antibody functionalized TiO_2_‐Chip exhibited markedly variable sEVs capture efficiency (79.00% ± 3.38%, 20.64% ± 4.57% and 55.46% ± 6.89%, respectively), which positively correlated with EpCAM expression levels (Figure S13), indicating inherent selection bias. These results indicated that the BAIP‐based strategy reduced dependence on antigen abundance compared with antibody‐antigen‐based capture, thereby enabling less biased isolation of sEVs across heterogeneous cell populations. We further evaluated nonspecific adsorption of albumin on the BAIP‐TiO_2_‐Chip. Given its high abundance in plasma (∼40 mg mL^−1^, accounting for ∼60% of total plasma protein), BSA solution at 70 and 1 mg mL^−1^ were used to represent high and low protein backgrounds, respectively. After incubation with the BAIP‐TiO_2_‐Chip, the residual BSA concentration remained essentially unchanged (Figure S15), indicating negligible albumin adsorption over a wide concentration.

### 2.3 Release and Functional Analysis of sEVs Captured on BAIP‐TiO_2_‐Chip

**FIGURE 3:**
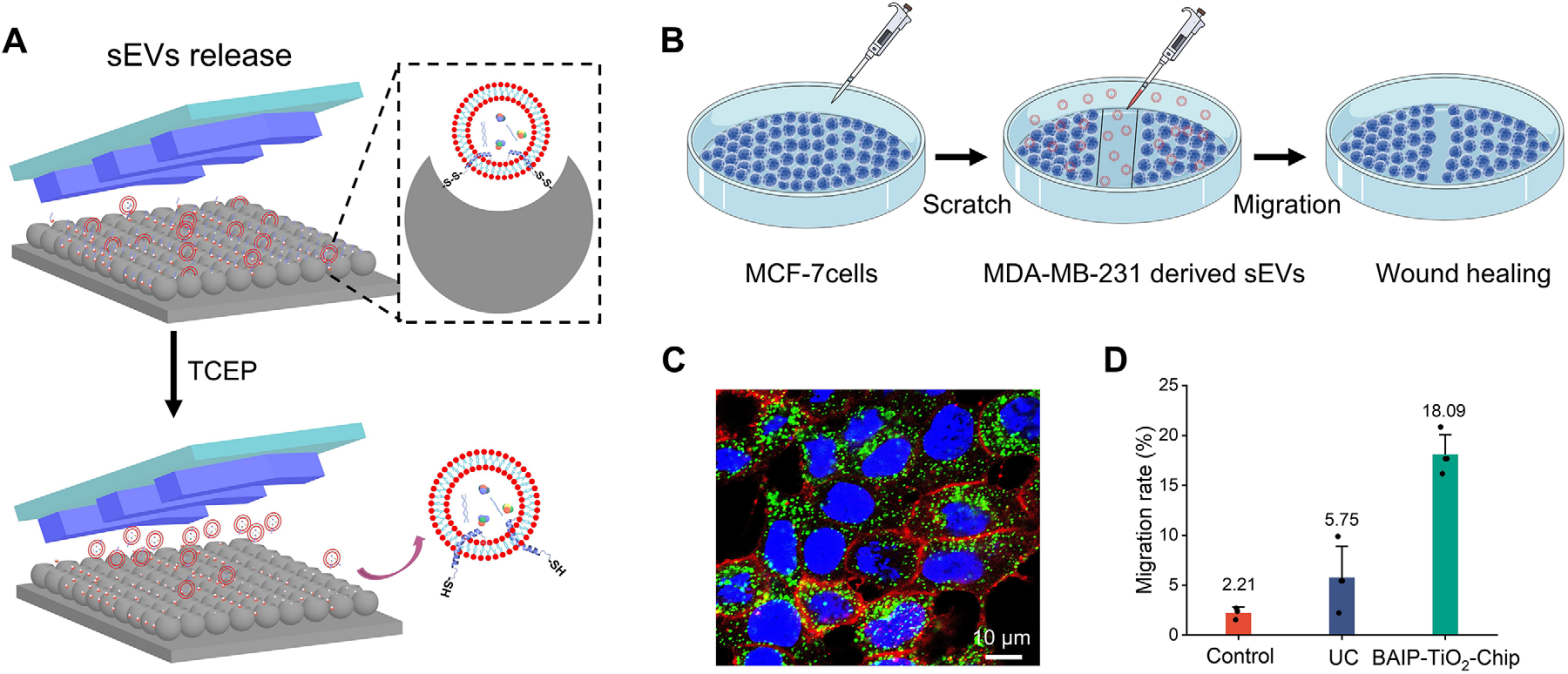
The release and functional analysis of sEVs. (A) Schematic diagram of the release of sEVs from BAIP‐TiO_2_‐Chip using TCEP. (B) Schematic diagram of wound healing assay. (C) Confocal image of MCF‐7 cells after incubating with MDA‐MB‐231 sEVs isolated by BAIP‐TiO_2_‐Chip for 5 h, blue, Hoechst 33342; green, EVs‐DiO; red, DiI. (D) Migration rate of MCF‐7 cells incubated with MDA‐MB‐231 sEVs isolated by UC and BAIP‐TiO_2_‐Chip, respectively.

In light of the emerging role of sEVs as targeted delivery vehicles in cancer therapy and as modulators in regenerative processes such as wound healing, establishing an efficient and controllable method for releasing captured sEVs was vital for preserving their therapeutic functionality and enabling clinical translation. To achieve this, the BAIP was replaced with a disulfide bond‐linked ssBAIP, enabling redox‐responsive release (Figure 3A). Upon treatment with tris (2‐carboxyethyl) phosphine (TCEP), 72.19% ± 7.09% of the sEVs captured on the ssBAIP‐TiO_2_‐Chip were released through the cleavage of disulfide bonds in ssBAIP (Figure S16). Then, the ssBAIP‐TiO_2_‐Chip was used to isolate sEVs from a 70 mg mL^−1^ BSA solution mimicking the high protein background of clinical plasma. The purity of the isolated sEVs was determined by the particle‐to‐protein ratio and benchmarked against ultracentrifugation. The results showed that the ssBAIP‐TiO_2_‐Chip achieved significantly higher purity than UC (Figure S17).

Given the mild release process and low toxicity of TCEP, the released sEVs were expected to retain their native biological activity. To verify this, cellular uptake and wound healing assays (Figure 3B) were performed using two types of breast cancer cells with distinct metastatic abilities (MCF‐7 cells characterized by low metastatic behavior and MDA‐MB‐231 cells by high metastatic propensity). To assess the functional integrity of the released sEVs, MDA‐MB‐231‐derived sEVs were labeled with DiO and incubated with MCF‐7 cells for 5 h. As shown in Figure 3C, the majority of the released sEVs were internalized by MCF‐7 cells, suggesting efficient uptake. This finding demonstrated that the TCEP‐mediated release process successfully preserved the functional integrity of the sEVs, thereby enabling efficient uptake by recipient cells.

To further assess the biological activity of the released sEVs, model sEVs from the highly aggressive MDA‐MB‐231 cells were isolated using two different methods: UC, serving as a conventional reference, and the BAIP‐TiO_2_‐Chip. These sEVs were then resuspended in serum‐free medium and introduced to low‐invasive MCF‐7 cells in a wound healing assay to examine their functional influence on cell migration. As shown in Figure 3D and Figure S18, MCF‐7 cells exhibited a migration rate of 18.09% ± 1.97% following treatment with sEVs released from the BAIP‐TiO_2_‐Chip, which was significantly higher than that of 5.75% ± 3.15% observed in the UC group. Furthermore, the same phenomenon was also observed when the amounts of sEVs isolated by the two methods were normalized to equal concentrations (Figure S19). These findings suggested that the TCEP‐triggered release from the BAIP‐TiO_2_‐Chip was more effective than UC in preserving the biological activity of sEVs. This advantage might stem from the gentler nature of our method compared to UC, which minimizes sEVs aggregation and breakage.

### 2.4 Proteomic Profiling of sEVs From Clinical Samples

Understanding the proteomic composition of sEVs was essential, as proteins mediate the vast majority of cellular signaling and functional processes. In early cancer diagnosis, analyzing EV‐associated proteins not only provides molecular insights into tumor biology but also offers valuable opportunities for clinical applications. Profiling established protein markers and discovering novel ones on sEVs contribute to early diagnosis, tumor classification, treatment monitoring, and prediction of therapeutic responses, particularly in the setting of immunotherapy [48, 49, 50, 51]. In particular, mass spectrometry (MS)‐based proteomic analysis of EVs facilitates the identification of tumor‐associated biomarkers and offers new insights into their biological roles through longitudinal and minimally invasive assessments [52, 53, 54]. However, obtaining high‐quality sEV samples had posed a major obstacle to reliable proteomic analysis for long time, primarily due to the presence of contaminating proteins and the limited efficiency of conventional isolation techniques [55]. Fortunately, this challenge might be effectively addressed by our BAIP based method [38].

**FIGURE 4:**
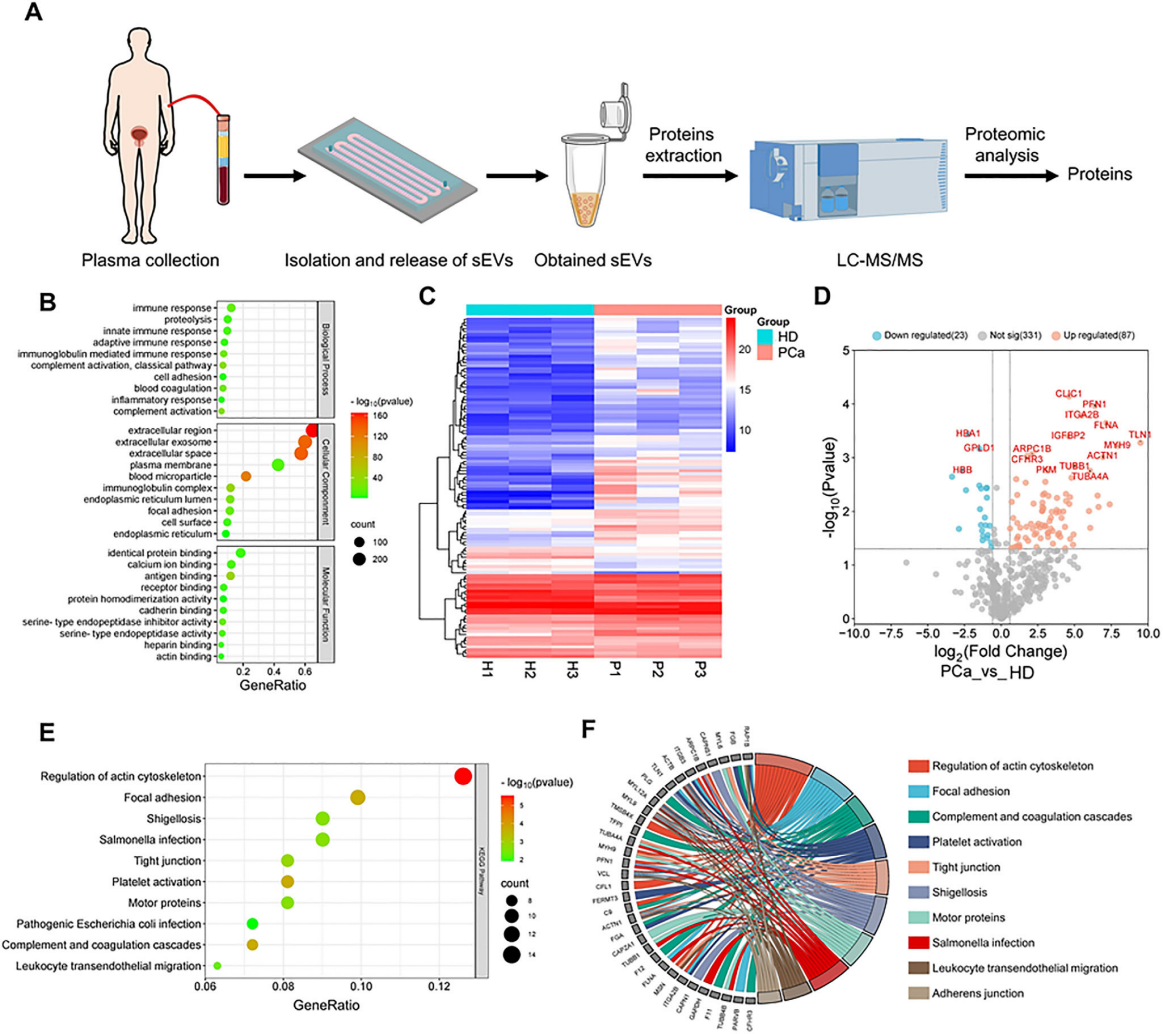
The proteomic profiling of sEVs. (A) Flowchart of sEVs protein profiling analysis in clinical samples. (B) GO enrichment analysis of biological process, cellular component, and molecular function on total proteins of PCa samples detected from more than half of the total samples. (C) Heatmap with differentially expressed proteins quantified from sEVs of PCa samples versus those from HD samples. (D) Volcano plot representing fold change of differentially expressed proteins quantified from sEVs of PCa samples versus those from HD samples. (E) Dot‐plot with significantly enriched KEGG pathways. (F) Network of genes of the differently expressed proteins linked to the KEGG pathway terms.

In order to obtain sufficient sEVs for proteomic profiling, we increased the yield of the BAIP‐TiO_2_‐Chip by linking five microfluidic chips in series (Figure S20). This configuration enabled the rapid processing of larger sample volumes and improved the overall yield of sEVs. The proteins released from the captured sEVs were initially analyzed by SDS‐PAGE, and the results confirmed that they were suitable for proteomic analysis (Figure S21). To further assess the potential clinical utility of this strategy, sEVs were isolated from the plasma of three PCa patients and three healthy donors (HD), and their protein profiles were compared as an exploratory analysis to identify candidate sEV‐associated proteins with differential expression (Figure 4A). The amount of sEV proteins detected in PCa samples was 19.05% higher than that in HD samples (Table S1). Gene Ontology (GO) analysis revealed that the major proteins identified in sEVs from clinical samples were characteristic of sEV‐related components and involved in a range of molecular functions, including identical protein binding, calcium ion binding, antigen binding, and receptor binding. These proteins were also involved in biological processes including immune response, proteolysis, complement activation, and inflammatory response (Figure 4B; Figures S22 and S23). Comparative proteomic analysis identified 110 differentially expressed proteins between PCa‐ and HD‐derived sEVs, among which 87 were significantly upregulated in the PCa group (Figure 4C,D). Further functional interaction analysis revealed that these proteins were significantly enriched in 26 KEGG pathways, including “regulation of actin cytoskeleton” and “focal adhesion” (Figure 4E,F). Collectively, these results highlighted the capability of the BAIP‐TiO_2_‐Chip for comprehensive sEV proteomic profiling and demonstrated its potential utility in clinical research for biomarker discovery and elucidating tumor‐associated molecular pathways.

### 2.5 Accurate Diagnosis of the PCa With Machine Learning Assisted mRNA Analysis

mRNA encapsulated within sEVs contained disease‐specific molecular signatures that offered valuable insights for early diagnosis, prognostic assessment, and the identification of novel therapeutic targets. In the context of PCa, although PSA testing was commonly employed as a first‐line screening tool, its diagnostic accuracy was limited, particularly for individuals with PSA levels in the so‐called “gray zone” (typically 4–10 ng mL^−1^ of PSA) [56, 57, 58]. Within this range, elevated PSA levels might arise from non‐malignant conditions such as benign prostatic hyperplasia, prostatitis, or other inflammatory responses, thereby reducing the specificity of the test. As a result, PSA testing alone often failed to clearly differentiate between benign and malignant prostate diseases, leading to diagnostic uncertainty and prompting many patients to undergo unnecessary and invasive procedures such as prostate biopsies. These limitations underscored the urgent need for more accurate and non‐invasive diagnostic strategies capable of supplementing or replacing PSA‐based screening [59]. In particular, sEV‐derived mRNA biomarkers held great promise for enhancing diagnostic precision, especially for patients with PSA levels in the ambiguous range [60, 61]. By providing a more reliable molecular profile of the disease state, such approaches might support more informed clinical decision‐making, reduce the number of avoidable biopsies, and ultimately improve the early detection and management of PCa.

**FIGURE 5:**
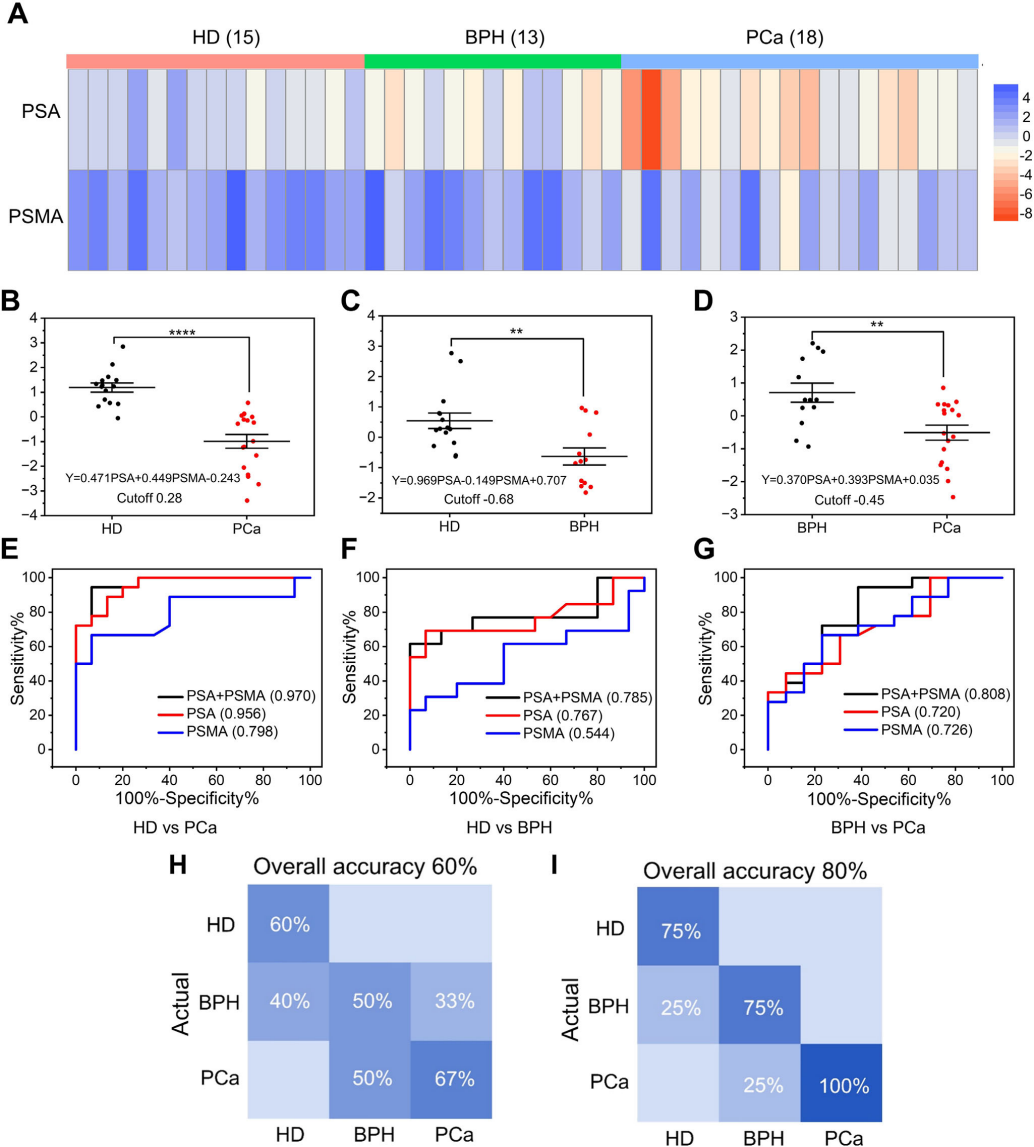
The mRNA analysis of sEVs. (A) Heatmap of the sEVs *PSA* and *PSMA* mRNA levels among the HD, BPH, and PCa. (B–D) The weighted sum of ΔCt of two markers by LDA for evaluating the discrimination power of HD and PCa (B), HD and BPH (C), BPH and PCa (D). (E–G) ROC analysis showing the performance of sEVs *PSA* mRNA, *PSMA* mRNA and combination of *PSA* and *PSMA* mRNA in distinction of HD and PCa (E), HD and BPH (F), BPH and PCa (G). (H) Confusion matrix based on ΔCt*_PSA_* and ΔCt*_PSMA_* summarizing the classification of HD, BPH, and PCa with 80% of the data allocated to the training set and 20% reserved for the prediction set. (I) Confusion matrix based on ΔCt*_PSA_*, ΔCt*_PSMA_*, sum (ΔCt*_PSA_* +ΔCt*_PSMA_*) and difference (ΔCt*_PSA_*−ΔCt*_PSMA_*) summarizing the classification of HD, BPH, and PCa with 80% of the data allocated to the training set and 20% reserved for the prediction set.

In this study, we aimed to detect the mRNA levels in sEVs to achieve accurate discrimination among PCa, benign prostatic hyperplasia (BPH), and HD. *PSA* and *PSMA* mRNA are highly correlated with prostate cancer. *GAPDH*, as a housekeeping gene, is used for data correction. As a preliminary step, we used model sEVs for method validation. Specifically, a 500 µL suspension of DU145 cell‐derived sEVs was captured using the BAIP‐TiO_2_‐Chips. The extracted total RNA was then subjected to RT‐qPCR analysis. Agarose gel electrophoresis and qPCR analysis confirmed the presence of specific PCR products corresponding to mRNA of *PSA*, *PSMA*, and *GAPDH* (Figures S24 and S25), indicating that our approach was capable of reliably detecting mRNA in sEVs. These results supported the potential applicability of this method for clinical transcriptomic analysis of sEVs. Subsequently, we used the BAIP‐TiO_2_‐Chip to rapidly and efficiently enrich sEVs from clinical plasma samples and to detect exocellular *PSA* and *PSMA* mRNA. This analysis was conducted on a cohort of 46 participants, comprising 18 patients with PCa, 13 with BPH, and 15 HD (Table S2). As depicted in Figure 5A and Figure S26, the ΔCt values (Ct*_PSA_*‐Ct*_GAPDH_* and Ct*_PSMA_*‐Ct*_GAPDH_*) in sEVs from PCa patients were significantly lower than those observed in BPH and HD groups, indicating higher expression levels of *PSA* and *PSMA* mRNA in the PCa cohort. In contrast, no statistically significant difference in either ΔCt*_PSA_* or ΔCt*_PSMA_* was detected between the HD and BPH groups.

To improve the diagnostic capacity of this mRNA‐based approach, we applied Linear Discriminant Analysis (LDA) to integrate the *PSA* and *PSMA* mRNA expression profiles across all samples. As shown in Figure 5B–D, the LDA revealed statistically significantly differences in discriminant scores across all pairwise comparisons, including PCa versus HD, BPH versus HD, and PCa versus BPH. The corresponding classification accuracies for these comparisons were 88%, 71%, and 68%, respectively (Figure S27), indicating good separation between PCa and HD, and moderate classification performance between BPH and the other two groups. To further evaluate the diagnostic performance, Receiver Operating Characteristic (ROC) curve analyses were conducted. The combined detection of PSA and PSMA mRNA demonstrated superior performance compared to either marker alone in distinguishing the three groups (Figure 5E–G). The areas under the curve (AUCs) for the combined analysis were 0.970 for PCa versus HD, 0.785 for BPH versus HD, and 0.808 for PCa versus BPH, highlighting the strong discriminatory power of the approach in identifying PCa cases from healthy individuals, while also enabling moderate separation of BPH from both HD and PCa. Taken together, these findings underscored the diagnostic potential of integrating multiple sEV‐derived mRNA markers for PCa detection. Nevertheless, the discriminative capacity between BPH and HD, as well as between PCa and BPH, was relatively modest. As such, they were insufficient for simultaneously achieving robust three‐way classification among PCa, BPH, and HD.

To further improve the diagnostic accuracy in distinguishing among HD, BPH, and PCa, a boosted decision tree classifier was developed using the XGBoost (eXtreme Gradient Boosting) software package. Prior to model training, 80% of the clinical samples were employed to extract input features based on ΔCt*_PSA_* and ΔCt*_PSMA_*. As shown in Figure 5H, the model achieved an overall accuracy of 60% on the held‐out test set, as visualized by the resulting confusion matrix. Notably, the model exhibited high accuracy in classifying HD and PCa samples but demonstrated limited performance in identifying BPH cases. To enhance model performance and provide the algorithm with additional decision‐making information, two additional features were generated: the arithmetic sum and difference of ΔCt*_PSA_* and ΔCt*_PSMA_*, resulting in a total of four input variables. Although these derived features lacked direct biological interpretation, they contributed to improved classification outcomes. The enhanced model achieved overall accuracies of 80% on the test set (Figure 5I), indicating a significant improvement of prediction over the initial configuration. These results collectively highlighted that expanding the number of input features, including those derived from engineered or composite variables, substantially enhanced model performance. These findings underscored that the BAIP‐TiO_2_‐Chip could serve as a non‐invasive and effective tool for PCa diagnosis. By enabling sensitive and specific mRNA profiling of *PSA* and *PSMA* from sEVs, the platform offers a promising approach to improve early diagnosis and differential classification of PCa‐related conditions.

## 3 Conclusion

In summary, we present a generalizable strategy for constructing high‐performance nanostructured interfaces for the isolation and molecular analysis of sEVs. By combining scalable electrospray fabrication of bowl‐shaped TiO_2_ nanospheres with a modular peptide‐based functionalization scheme, we engineered a three‐dimensional high‐curvature interface capable of topology‐ and affinity‐selective capture of sEVs. Integration with herringbone microfluidic structures enhanced analyte‐surface interactions and collision frequency, enabling rapid, efficient, and reproducible sEV enrichment. The BAIP‐TiO_2_‐Chip achieved a capture efficiency exceeding 90% within 5 min. Compared with existing methods (Table S3), our method exhibited significantly faster capture kinetics and higher capture efficiency. The platform also supports redox‐responsive release of intact sEVs and downstream molecular analyses, including proteomic and mRNA profiling. Proteomic analysis revealed differentially expressed proteins between HD and PCa patients, providing insights into tumor‐associated molecular alterations. In parallel, mRNA detection of *PSA* and *PSMA* within sEVs, combined with a boosted decision tree model, achieved a diagnostic accuracy of 80% on the test set for distinguishing PCa, BPH, and HD. Beyond PCa, the BAIP‐TiO_2_‐Chip offers a broadly applicable approach for sEV isolation and multi‐omics profiling, with significant potential in liquid biopsy, biomarker discovery, and disease monitoring.

## 4 Experimental Section

### 4.1 Reagents and Materials

Tetra‐n‐butyl titanate was purchased from Sinopharm (Shanghai, China). BSA and polyvinylpyrrolidone (PVP, MW 58 000) were purchased from Aladdin (Shanghai, China). Streptavidin (SA) and sulfosuccinimidyl 6‐(biotinamido) hexanoate (Sulfo‐NHS‐LC‐Biotin) were purchased from APExBIO (Shanghai, China). Polydimethylsiloxane (PDMS) was purchased from GE Toshiba Silicones Co. Ltd. (Tokyo, Japan). Mouse anti‐human CD9 antibody was purchased from Biolegend, Inc. (Beijing, China). Cy3‐IgG was purchased from Boster Biological Technology Co. Ltd (Wuhan, China). SA‐Cy3 was purchased from Sangon Biotech Co., Ltd (Shanghai, China). Dulbecco's modified Eagle's medium (DMEM, Gibco) and Fetal bovine serum (FBS, Gibco) were purchased from Thermo Fisher Scientific Inc. (Shanghai, China). The BAIP was synthesized by Motif Biotech (Motif Biotechnology Co., Ltd, Suzhou, China) as a previous report [38]. Deionized water was obtained by Hitech‐Kflow Water purification system (Shanghai, China). Phosphate buffered saline with Tween‐20 (PBST) is PBS with the addition of 0.05% Tween 20. All oligonucleotides were synthesized by Sangon Biotech Co., Ltd.

### 4.2 Electrospray of Bowl‐Shaped TiO_2_ Nanospheres on the Glass Slides

Bowl‐shaped TiO_2_ nanospheres were synthesized via electrospray technology as illustrated in Figure S1. PVP (0.9 g) was added into 3 mL of ethanol solution under vigorous stirring for about 30 min to obtain PVP solution. Another solution, prepared by mixing 1.5 g of tetra‐n‐butyl titanate, 3 mL of ethanol and 3 mL of acetic acid with magnetically stirring for 10 min, was then mixed with the PVP solution and magnetically stirred for another 1 h to obtain the TiO_2_ precursor solution. The TiO_2_ precursor solution was sucked into 1 mL syringe, which was set on an injection pump with an outflow speed of 0.5 mL h^−1^. Meanwhile, the working voltage was controlled as 10 kV, the distance between receiver (75 × 25 mm glass slides) and the syringe needle was adjusted to 10 cm. After electro‐spraying for a certain time, the coated glass slides were dried in vacuum at 120°C for 12 h, calcined in muffle furnace at 450°C for 3 h and then naturally cooled down to room temperature. The bowl‐shaped TiO_2_ nanospheres modified glass slides were obtained.

### 4.3 The Fabrication of TiO_2_‐Integrated Microfluidic Chip

The herringbone structure and the PDMS microchannel were fabricated by standard photolithography technology as a published work [62]. Briefly, SU‐8 10 photoresist was spin‐coated on a silicon wafer to create a master consisting of two‐layer features of both 25 µm by a spin coater (Jiatu‐Tech, EZ6, China). The upper layer of the feature formed the herringbone structures which had five ridges in one cycle with a groove width of 66.6 µm, groove pitch of 133.4 µm, and an angle between herringbone channel of 45°. The PDMS module was fabricated by pouring PDMS prepolymer (10:1 of RTV615A monomer/RTV615B catalyst, w/w) into the silicon mold and then cured in a 75°C oven for 4 h after degassing. Afterward, the solid PDMS replicas were peeled off from the silicon mold and punched with a blunt needle to form inlet and outlet. Finally, the bowl‐shaped TiO_2_ nanospheres‐integrated microarray chip (TiO_2_‐Chip) was assembled by using the “stamp‐stick‐adhesion” strategy as a published work [62]. For the comparison, PDMS module was bonded to the bare glass slides without TiO_2_ coating (Bare‐Chip).

### 4.4 The Modification of BAIP on TiO_2_‐Chip

First, 50 µL of the synthesized BSA‐biotin (100 µg mL^−1^), which was obtained according to a previous report [63], was pumped into TiO_2_‐Chip at 10 µL min^−1^ and incubated for 30 min. After being washed with triploid PBST, 50 µL of SA solution with a certain concentration was pumped into the chip at 10 µL min^−1^ and incubated for 30 min. After being washed with triploid PBST, 50 µL of BAIP with a certain concentration was pumped into the chip at the same flow rate and incubated for 30 min. After being blocked with 5% BSA and completely cleaned, the BAIP modified TiO_2_‐Chip (BAIP‐TiO_2_‐Chip) was stored at 4°C and used as soon as possible. During this process, the concentrations of SA and BAIP were optimized. For optimization of SA concentration, we replaced SA with SA‐Cy3 to make quantification possible by fluorescence intensity (λ_ex_ = 520 nm and λ_em_ = 570 nm). The SA‐Cy3 solution was measured by an F‐4600 fluorescence spectrophotometer (Hitachi, Japan) before and after incubation with the TiO_2_‐Chip for 30 min. For the modification of BAIP, the BAIP solution was measured before and after incubation with the TiO_2_‐Chip by Nanodrop 2000C (Thermo, USA) to calculate the mass of BAIP. The grafting density (*ρ*, molecules cm^−2^) of the BAIP was calculated according to Equation (1)

(1) $$\rho =\frac{\left(C_{0}-C_{e}\right)VN_{A}}{M_{w}S}\times 10^{-9}$$

where *C_0_* (ng µL^−1^) is the initial concentration of BAIP, *C_e_* (ng µL^−1^) is the concentration of remaining BAIP after incubation, *V* (µL) is the volume of BAIP solution, *N_A_* is 6.022 × 10^23^ mol^−1^, *M_W_* is 4668.1 g mol^−1^, and *S* (cm^2^) is the area of the microchannel of TiO_2_‐Chip.

### 4.5 Fluid Simulation

The fluid behavior within the microchannel was analyzed using COMSOL Multiphysics 6.2 finite element simulation software. Model setup: TiO_2_ with a diameter of 800 nm and a depression depth of 200 nm was simulated under a fluid flow rate of 10 µL min^−1^ (equivalent to a linear velocity of approximately 1.8 mm s^−1^ in a microfluidic channel with a width of 1 800 µm and a height of 50 µm). The TiO_2_ depressions were arranged along the Z‐axis within the microchannel. An “incompressible fluid/steady‐state” finite element model was selected. A part of the channel was simulated, with TiO_2_ introduced in a confined region at the front end of the chip to analyze their localized impact on flow velocity.

### 4.6 Cell Culture

MCF‐7 cells (RRID: CVCL_0031) were purchased from the Cell Bank of the Chinese Academy of Sciences (Shanghai, China). MDA‐MB‐231 cells (RRID: CVCL_0062) and DU145 cells (RRID: CVCL_0105) were purchased from ProCell (Wuhan, China). They were confirmed to be free of contamination and cultured in DMEM plus 10% FBS and 1% penicillin‐streptomycin in an incubator containing 5% CO_2_ at 37°C.

### 4.7 Isolation and Characterization of Model sEVs

The cells were cultured in FBS‐free media for 48 h until cells reached 80% ∼ 90% confluency. The culture media were collected and centrifuged at 4°C to remove cells and cellular debris (2 000 × *g*, 10 min), and the resulting supernatant was centrifuged at 10 000 × *g* for 30 min at 4°C to remove microvesicles, followed by ultracentrifugation at 120 000 g for 85 min at 4°C using a type 70 Ti rotor in an Optima XE‐100 ultracentrifuge (Beckman Coulter). The resulting sEVs were resuspended and washed with sterile PBS, followed by another ultracentrifugation at 120 000 g for 85 min at 4°C. The obtained sEVs were resuspended in sterile PBS and stored at −80°C for further use. The TEM, NTA, and Western Blot characterizations of sEVs were carried out based on a previous report [38].

### 4.8 Collection of Clinical Samples

Clinical samples of healthy person and cancer patients were obtained from Tongji Hospital of Huazhong University of Science and Technology (Wuhan, China), which was approved by the ethics committees of Tongji Hospital of Huazhong University of Science and Technology ([2024] IEC(A093)). Participants provided informed consent prior to sample collection with accompanying clinical information (Table S2). In the clinical cohorts, the HD (*n* = 15), the BPH (*n* = 13), and the PCa (*n* = 18) were collected. The blood samples were centrifuged at 3 000 × *g* for 10 min to remove cells and obtain cell‐free plasma. Then, the plasma was centrifuged at 10 000 × *g* at 4°C for 30 min to remove large vesicles. All samples were processed on the day of collection. After processing, samples were either used within 1 h or stored at −80°C, and used within 24 h to minimize potential variability.

### 4.9 Isolation of sEVs Using BAIP‐TiO_2_‐Chip

Model sEVs (∼8.3 × 10^10^ particles mL^−1^, 50 µL) were pumped into the BAIP‐TiO_2_‐Chip with a certain flow rate and incubated for a certain time. After incubation, the uncaptured sEVs were pumped out with air. The protein contents of the residual sEVs and initial sEVs were measured by Micro BCA Protein Assay Kit (Sangon Biotech, China) according to the manufacturer's instructions. The capture efficiency (*η_c_*) of sEVs was calculated according to Equation (2).

(2) $$\eta_{c}=\frac{C_{0}-C_{e}}{C_{0}}\times 100\%$$

where *C_0_* (µg mL^−1^) is the protein contents of initial sEVs, *C_e_* (µg mL^−1^) is the protein contents of residual sEVs after capture. For the comparison, Bare‐Chip, TiO_2_‐Chip, and BAIP‐TiO_2_‐Chip were incubated with the same model sEVs, the capture efficiencies of sEVs were calculated and compared. All experiments were repeated three times.

### 4.10 Immuno‐Fluorescence Staining

After capture of sEVs, the BAIP‐TiO_2_‐Chip was thoroughly washed with PBS. Then, the sEVs‐captured BAIP‐TiO_2_‐Chip was incubated with 10 µg mL^−1^ mouse anti‐human CD9 antibody for 1.5 h at room temperature. After washed and blocked with 5% BSA, the BAIP‐TiO_2_‐Chip was then incubated with 10 µg mL^−1^ Cy3‐IgG for 30 min. After throughly cleaned, the labelled sEVs‐captured BAIP‐TiO_2_‐Chip was observed under an inverted fluorescence microscope. For the control group, the experiment was the same as above without capturing of sEVs.

### 4.11 Release of the Captured sEVs from BAIP‐TiO_2_‐Chip

Similar to the previous report [38], to release the captured sEVs, disulfide bond modified BAIP (ssBAIP) instead of BAIP was used to generate ssBAIP‐TiO_2_‐Chip. After sEVs capture, 50 µL of 1 mM Tris(2‐carboxyethyl)phosphine (TCEP) was pumped into ssBAIP‐TiO_2_‐Chip and incubated for 1 h and then pumped out with air. The concentrations of the initial sEVs, the residual sEVs and the released sEVs were quantified using NanoFCM (N30E, China). The released efficiency (*η_r_*) of sEVs was calculated according to Equation (3).

(3) $$\eta_{r}=\frac{C_{r}}{C_{0}-C_{e}}\times 100\%$$

where *C_0_* (particles mL^−1^) is the concentration of initial sEVs, *C_e_* (particles mL^−1^) is the concentration of residual sEVs after capture and *C_r_* (particles mL^−1^) is the concentration of released sEVs.

### 4.12 Cell Uptake of sEVs

DiO (10 µM) was used to prestain 300 µL of the MDA‐MB‐231 cell derived model sEVs and the excess DiO was removed using ultrafiltration (100 kDa). The sEVs were resuspended in 300 µL of PBS. Then, the sEVs were re‐enriched with ssBAIP‐TiO_2_‐Chip and released with TCEP. Approximately 5 × 10^5^ MCF‐7 cells were seeded into confocal dishes and incubated overnight. After MCF‐7 cells cocultured with the released sEVs for 5 h at 37°C, the medium was removed and replaced with Hoechst 33342 (10 µM) and DiI (10 µm) at 37°C for 30 min. Next, the medium was discarded, and the cells were washed with PBS. The cells were observed under a confocal fluorescence microscope equipped with a 100× oil lens at the respective emission and excitation wavelengths of individual dyes, i.e. Hoechst 33342 (λ_ex_ = 350 nm, λ_em_ = 430 to 475 nm), DiI (λ_ex_ = 561 nm, λ_em_ = 570 to 616 nm) and DiO (λ_ex_ = 486 nm, λ_em_ = 500 to 550 nm).

### 4.13 Wounding Healing Assay

The solution (1 800 µL) of model sEVs derived from the highly aggressive MDA‐MB‐231 cells was divided into six parts, and each three parts of sEVs were re‐enriched with ssBAIP‐TiO_2_‐Chip and UC, respectively. The sEVs‐bound ssBAIP‐TiO_2_‐Chips were incubated with TCEP to release the sEVs, and momently stored at 4°C. The sEVs of UC were resuspended in 900 µL of DMEM and momently stored at 4°C. Approximately 1 × 10^5^ of MCF‐7 cells were seeded into a 48‐well plate. When the cells reached ∼90% confluence, they were scratched by a 10 µL pipette tip. The culture medium was discarded and washed with PBS three times to remove residual sEVs and FBS from the medium. MCF‐7 cells cocultured with the above sEVs containing in the DMEM medium, and the wound width was measured under microscope at 0 h and 24 h.

### 4.14 RNA Extraction and qRT‐PCR

After isolation by BAIP‐TiO_2_‐Chip, the sEVs in clinical samples were lysed with NucleoZol (catalog#740404.200, Genecompany, China) according to a previous report [38]. The subsequent cDNA conversion was conducted using Plus All‐in‐one first Strand cDNA Synthesis SuperMix (catalog#E047, NovoScript, China) according to the manufacturer's instructions. qPCR assays were then performed using the resulted cDNA for the quantification of the 428 bp of the coding region of *PSA* gene, the 196 bp of the coding region of *PSMA* gene and the 300 bp of the coding region of *GAPDH* gene on a real‐time PCR detection system (Roche LightCycler 96). *GADPH* mRNA was chosen as the house keeping gene, and primers used for each tested gene were listed in Table S4. The qPCR was performed in 25 µL reaction system containing 12.5 µL of Premix Ex Taq Hot Start Version (catalog#RR030A, Takara, China), 2 µL of cDNA, 0.75 µL of each primer, 1.25 µL of Evagreen and 7.75 µL of ddH_2_O. Amplification followed the thermal profile of 94°C for 3 min, 50 cycles of 94°C for 30 s, 60°C for 30 s, 72°C for 30 s, and a final melting.

### 4.15 Protein Extraction and Label Free Quantification Proteomic Profiling of the Isolated sEVs

The sEVs in clinical samples from PCa and healthy donors were isolated by BAIP‐TiO_2_‐Chips and then lysed with RIPA buffer containing cocktail protease inhibitors on ice for 4 h. The lysates were pumped out and centrifuged at 12 000 g for 8 min at 4°C and the concentration of protein in supernatant was determined using BCA Protein Assay Kit (catalog#C503021, Sangon Biotech Co., Ltd, China). The same amount of protein was reduced with TCEP and alkylated with iodoacetamide at 37°C for 1 h, then digested with trypsin (1:50, w/w) overnight at 37°C and terminated by trifluoroacetic acid. After centrifuged and desalted, the peptides were vacuum dried and stored at −20°C for later use.

4D label‐free quantitative proteomic profiling was performed. The peptides were resuspended and analyzed on timsTOF Pro (Bruker Daltonics) and UltiMate 3000 RSLCnano system (Thermo Fisher Scientific). Peptide samples were separated in a C18 column (75 µm×15 cm, 1.7 µm, 100 Å) with a flow rate of 300 nL min^−1^. The MS raw data were processed with MaxQuant (version 2.0.1.0) against the human protein sequence database download from UniProt (20230619, 20423 entries) using the following parameters: Enzyme digestion specificity was set to Trypsin/P with maximum 2 missed cleavages; Peptide mass tolerance for initial precursor ions was set at 20 ppm in first search and 10 ppm in main search; False discovery rate was set at 1%. The relative quantitative value was obtained according to LFQ intensity between each sample: The proteins were deemed to be upregulated when FC > 1.5. The proteins were deemed to be downregulated when FC < 1.5. KEGG pathways and Gene Ontology (GO) analysis were performed using DAVID (https://david.ncifcrf.gov/home.jsp). The threshold for q‐value was set at 0.05. Fold changes were calculated by making a patient/normal ratio of LFQ intensity and log2 transforming these values.

### 4.16 Statistical Analysis

We used Origin 9.0 and SPSS 27 for statistical analyses and graphical representation. Data were represented as mean ± standard deviation for at least three replicates. Statistical analyses were assessed by two‐tailed t test and one‐way ANOVA with significance set at *p* < 0.05. The performance of BAIP‐TiO_2_‐Chip in PCa diagnosis was evaluated using receiver operating characteristic (ROC) analysis and linear discriminant analysis (LDA).

## Author Contributions

L.X., X.Y., Z.W. and L.W. conceived the idea and designed the experiment. L.W. and Y.L. performed the experiments and collected data. M.S. collected a clinical sample. J.D. performed the machine learning (XGBoost). L.W. conducted the microfluidic chip design and bioinformatics analysis. L.X., X.Y., M.J. and L.W. wrote the original manuscript. All authors read and approved the manuscript.

## Conflicts of Interest

The authors declare no conflicts of interest.

## Supporting information




**Supporting File**: advs75699‐sup‐0001‐SuppMat.docx.

## Data Availability

The data that support the findings of this study are available from the corresponding author upon reasonable request.
