# Supplementary material for: A Bioinspired Three‐Dimensional High‐Curvature Nano‐Interface Integrated Microfluidic Chip for Small Extracellular Vesicles Enrichment and Machine Learning‐Assisted Prostate Cancer Precision Diagnosis
Source: Adv Sci (Weinh). 2026 May 14:e21178. Online ahead of print. doi: 10.1002/advs.202521178 (PMC13335776; doi:10.1002/advs.202521178)
Supplement: Supplementary file 1 — Supporting File: advs75699‐sup‐0001‐SuppMat.docx. [file ADVS-9999-e21178-s001.docx]

Supporting Information

A Bioinspired Three-Dimensional High-Curvature Nano-Interface Integrated Microfluidic Chip for Small Extracellular Vesicles Enrichment and Machine Learning-Assisted Prostate Cancer Precision Diagnosis

Le Wang,^1,2^^†^ Yizhong Liang,^2†^ Manan Sulaiman,^3^ Jiaqi Du,^2^ Ming Jiang,^2^ Zhihua Wang,^3^* Xu Yu,^2^* and Li Xu^2^*

^1^L. Wang

Department of Pharmacy, Tongji Hospital, Tongji Medical College, Huazhong University of Science and Technology, Wuhan 430030, China

^2^L. Wang, Y. Liang, J. Du, M. Jiang, X. Yu, L. Xu

Tongji School of Pharmacy, Huazhong University of Science and Technology, Wuhan 430030, China

E-mail: xuyu@hust.edu.cn; xulpharm@mails.tjmu.edu.cn

^3^M. Sulaiman, Z. Wang

Department of Urology, Tongji Hospital, Tongji Medical College, Huazhong University of Science and Technology, Wuhan 430030, China

E-mail: zhwang_hust@hotmail.com

^†^These authors contribute equally to this study.


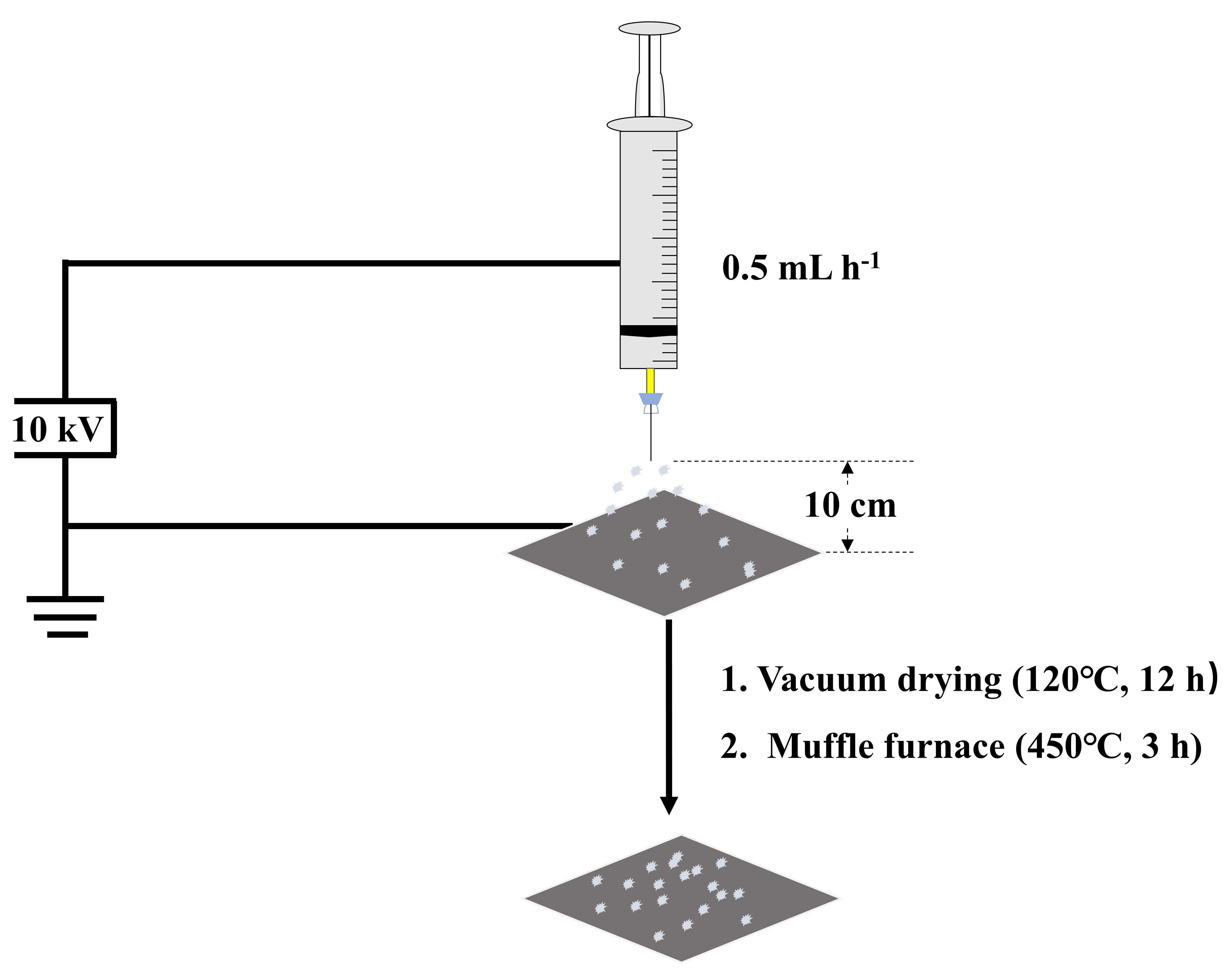


Figure S1. The schematic diagram for synthesis of bowl-shaped TiO_2_ nanospheres interface.


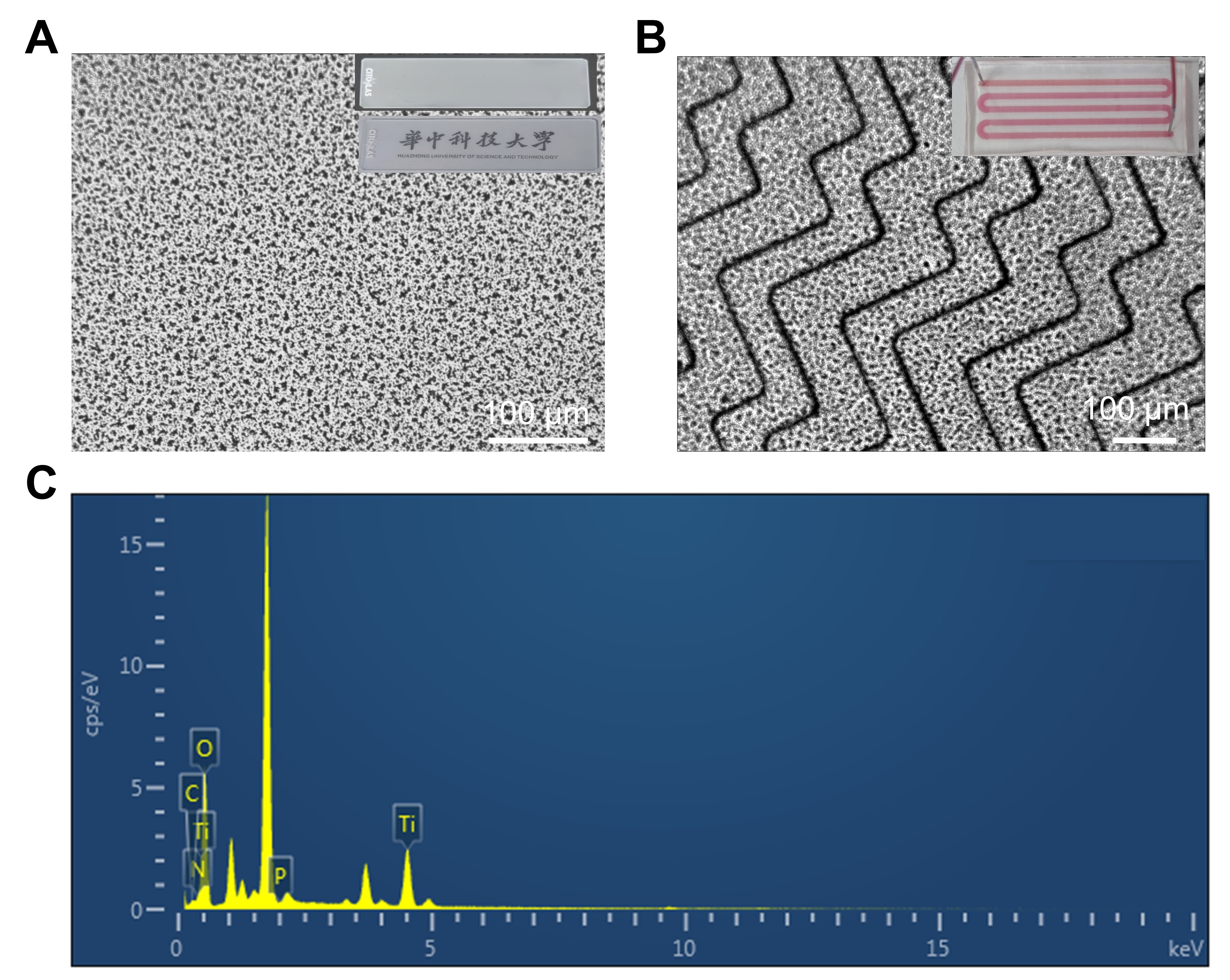


Figure S2. A) Microscopic image and optical photograph of the 3D Hic-TiO_2_ interface. B) Microscopic image and optical photograph of TiO_2_-Chip with herringbone structure. C) Elemental analysis of 3D Hic-TiO_2_ using field emission scanning electron microscopy-energy dispersive spectrometer.


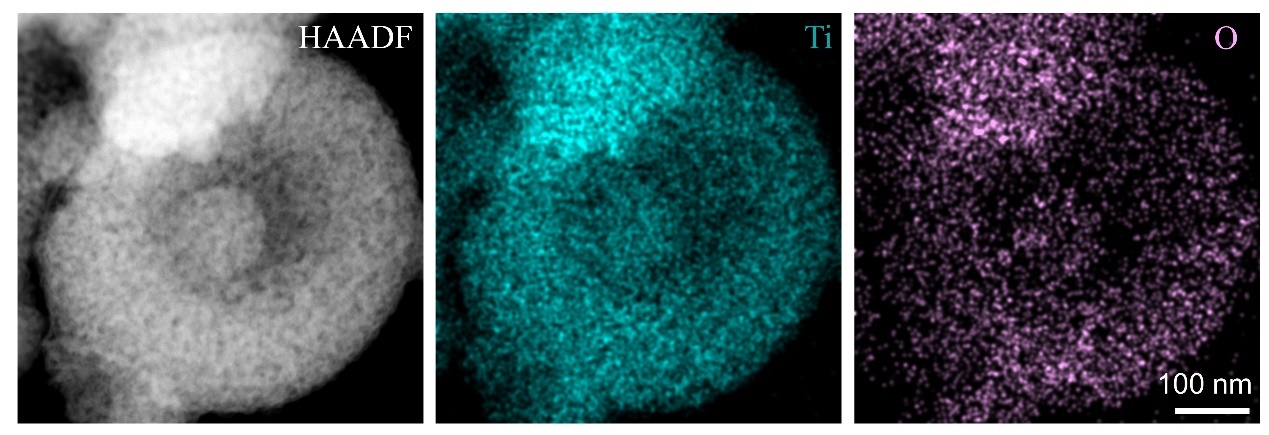


Figure S3. TEM images of 3D Hic-TiO_2_ with corresponding elemental mapping of Ti and O.


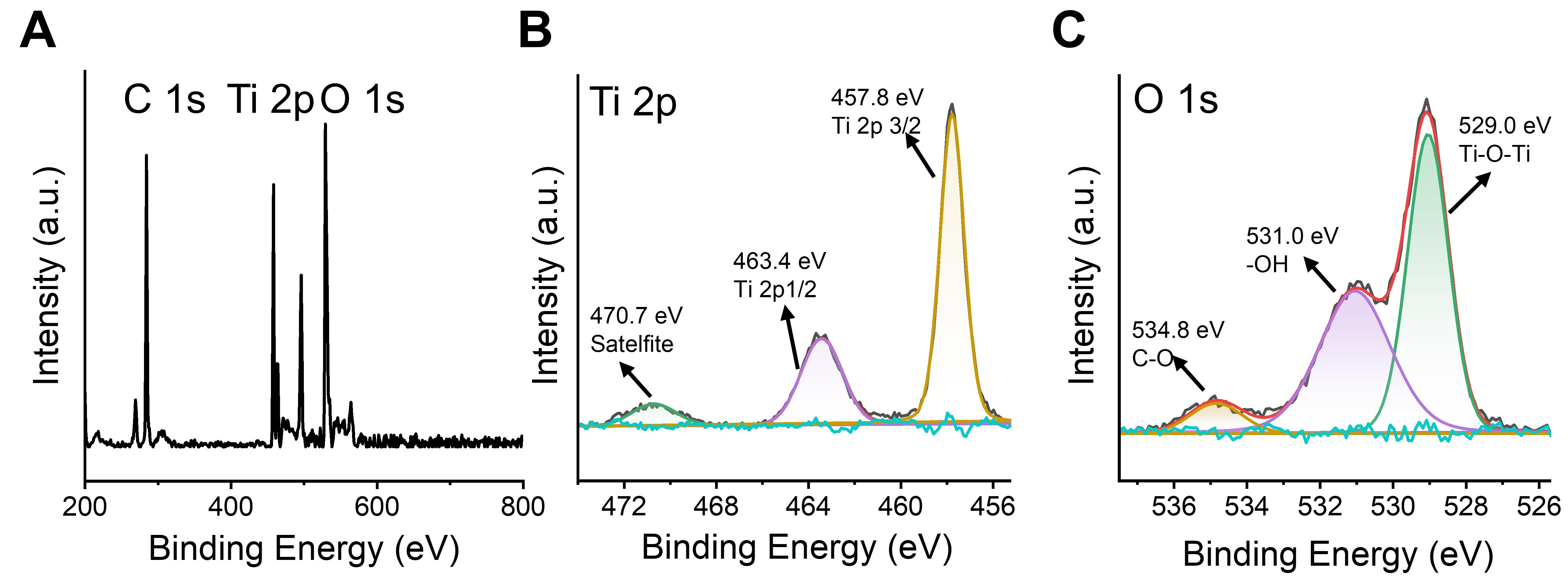


Figure S4. XPS analysis of 3D Hic-TiO_2_. The full scan spectrum A), high-resolution spectra of Ti 2p B) and O 1s C).


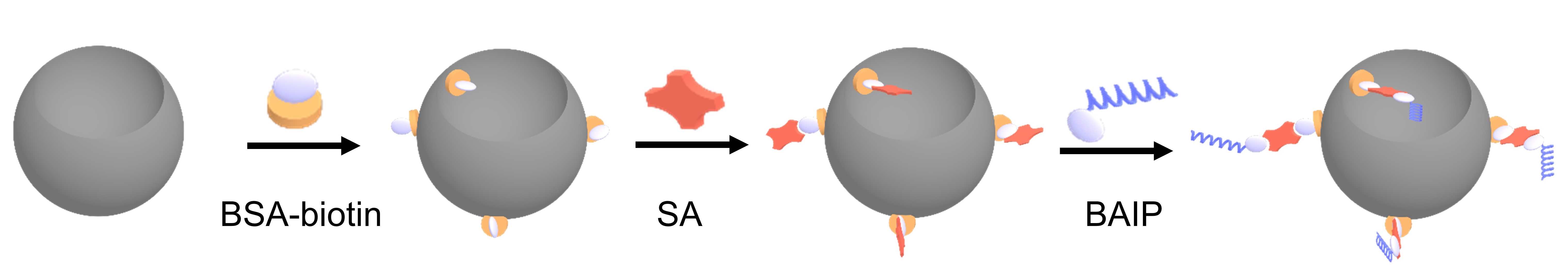


Figure S5. The scheme of BAIP modification on TiO_2_-Chip.


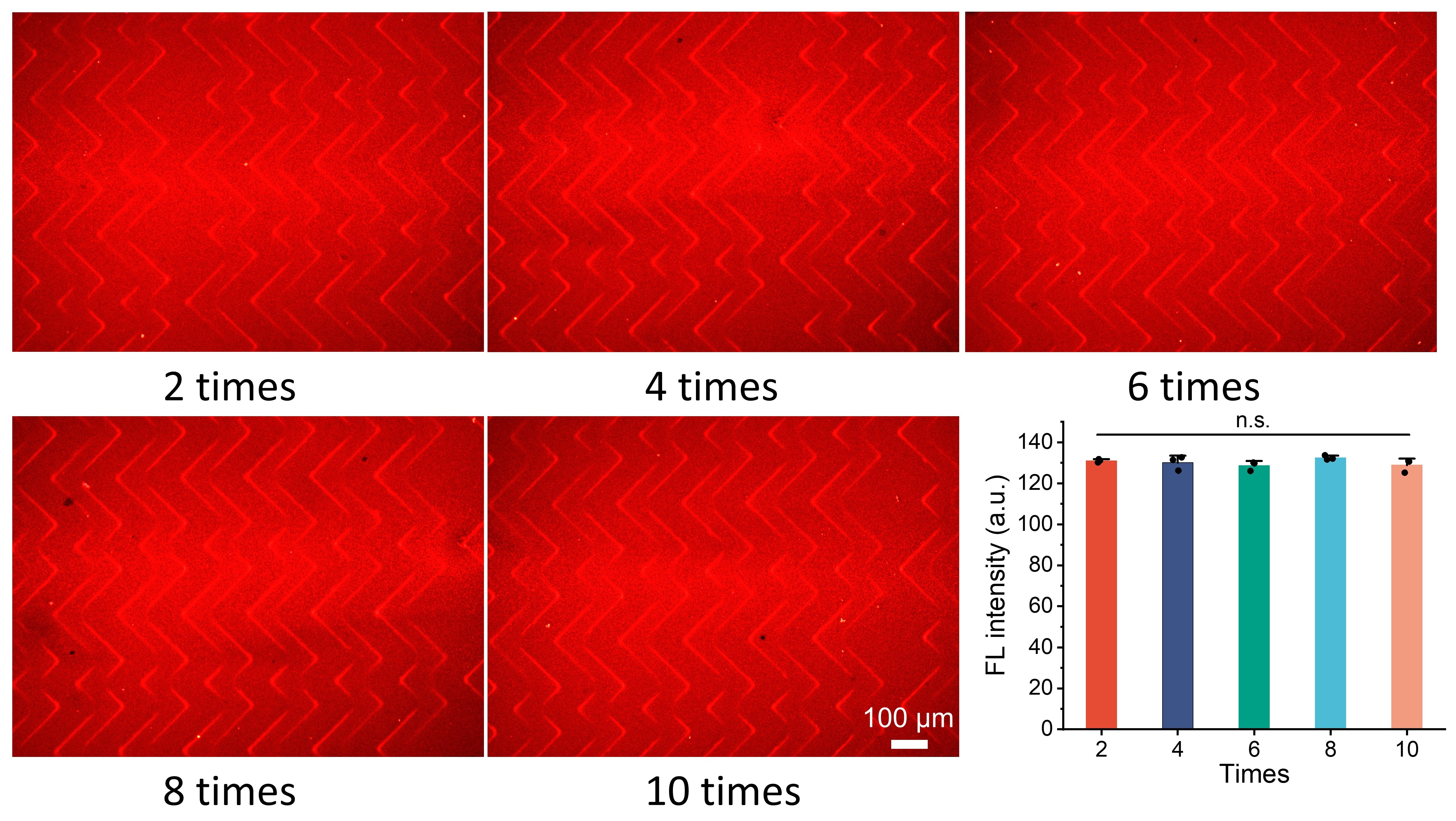


Figure S6. Microscopic fluorescence images of TiO_2_-Chip. The stability of BSA-biotin modified on TiO_2_-Chip was investigated with SA-Cy3. The fluorescence intensity of Cy3 was calculated by Image J software. During 10 times wash, the fluorescence intensity of Cy3 kept stable.


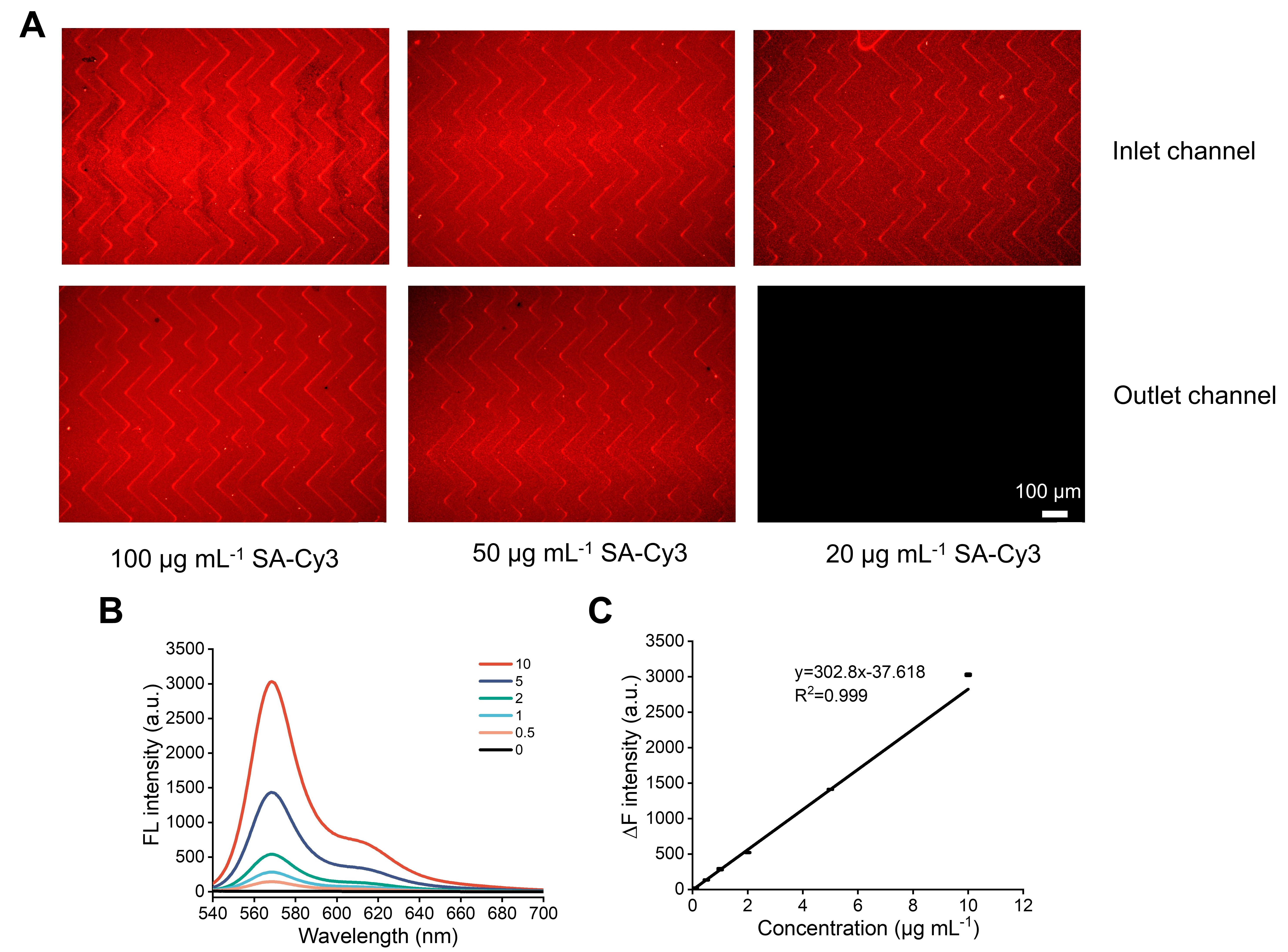


Figure S7. SA-Cy3 was used instead of SA to characterize SA modification. A) Microscopic fluorescence images of BAIP-TiO_2_-Chip. When the concentration of SA was 20 μg mL^-1^, only the inlet channel emitted fluorescence, while the outlet channel did not, indicating that the amount of SA was insufficient to modify the entire channel. When increasing the concentration of SA to 50 μg mL^-1^ and 100 μg mL^-1^, the amount of SA was sufficient to modify the entire channel. B) Fluorescence spectra of SA-Cy3 with concentration from 0 to 10 μg mL^-1^. C) Linear plot of the fluorescence intensity versus SA-Cy3 concentration.


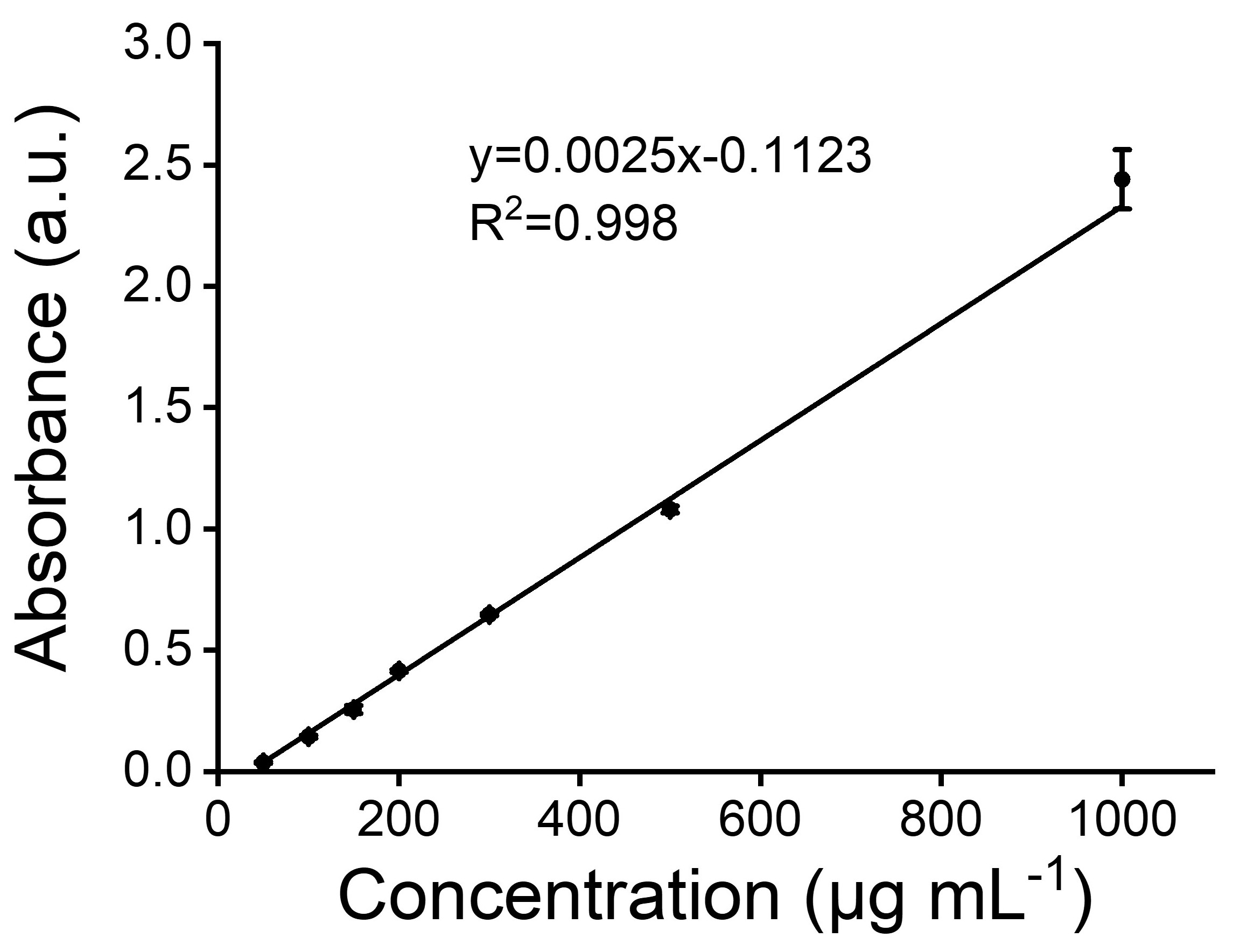


Figure S8. Linear plot of the BAIP concentration versus absorbance measured by Nanodrop.


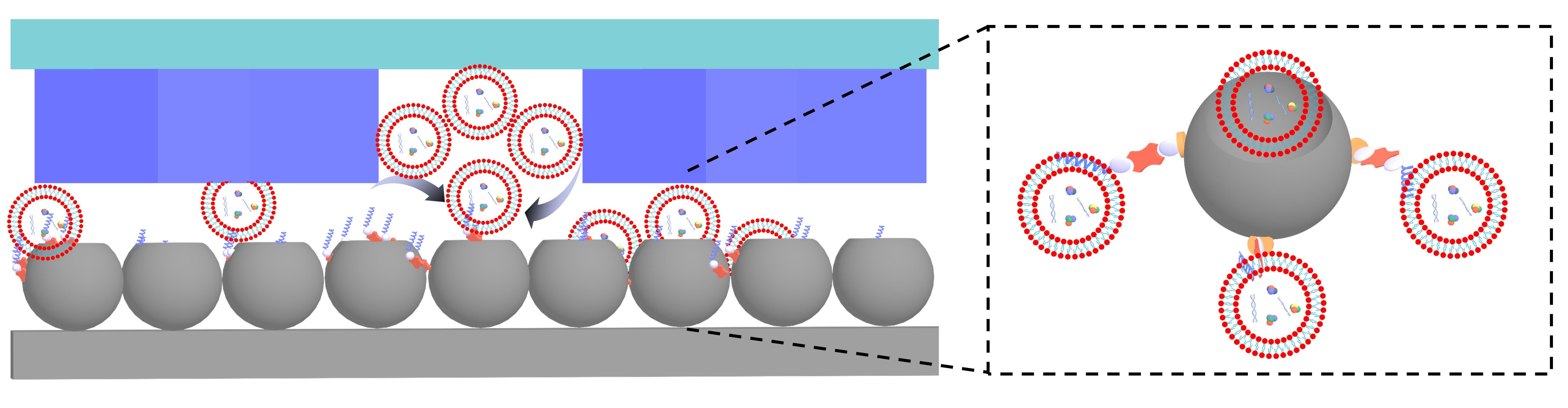


Figure S9. The scheme of efficient capture of sEVs through affinity interaction, topological size recognition, and the enhanced collision between sEVs and the BAIP-functionalized interface on the microfluidic chip.


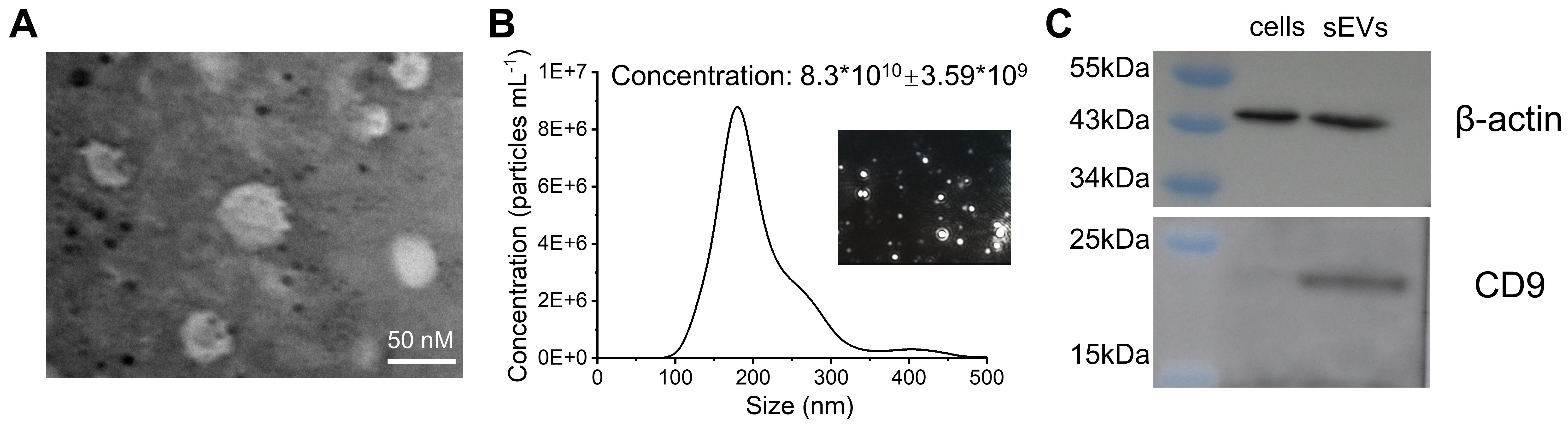


Figure S10. Characterization of model sEVs. A) The TEM image of sEVs derived from MCF-7 cells. B) NTA characterization of sEVs isolated from MCF-7 cells. The result showed that the average size of sEVs was 207.8 nm and the concentration was approximately 8.30 × 10^10^ particles mL^-1^. C) Western blot analysis of MCF-7 cells and model sEVs. The presence of membrane protein CD9 and the reference protein β-actin were successfully identified. Under equivalent β-actin loading conditions, the CD9 protein band in sEVs exhibited greater intensity than that in their parent cells, demonstrating that CD9 is significantly enriched in sEVs.


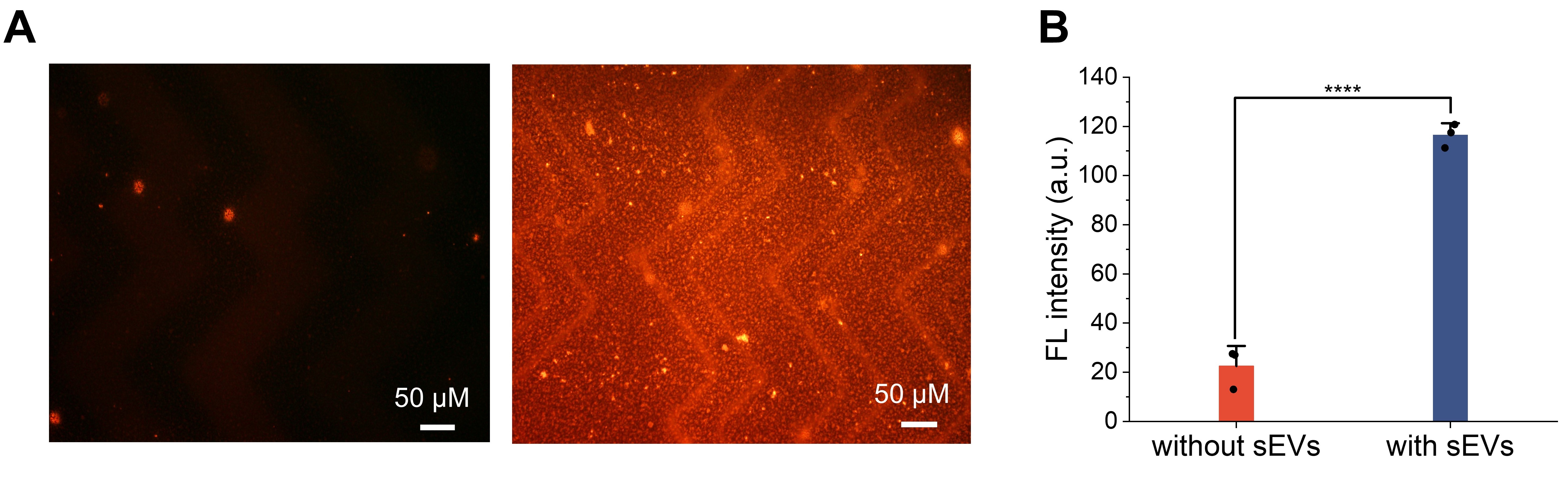


Figure S11. Fluorescence signal intensities of CD9 protein for blank sample and MCF-7 sEVs captured with BAIP-TiO_2_-Chip. *****P* < 0.0001.


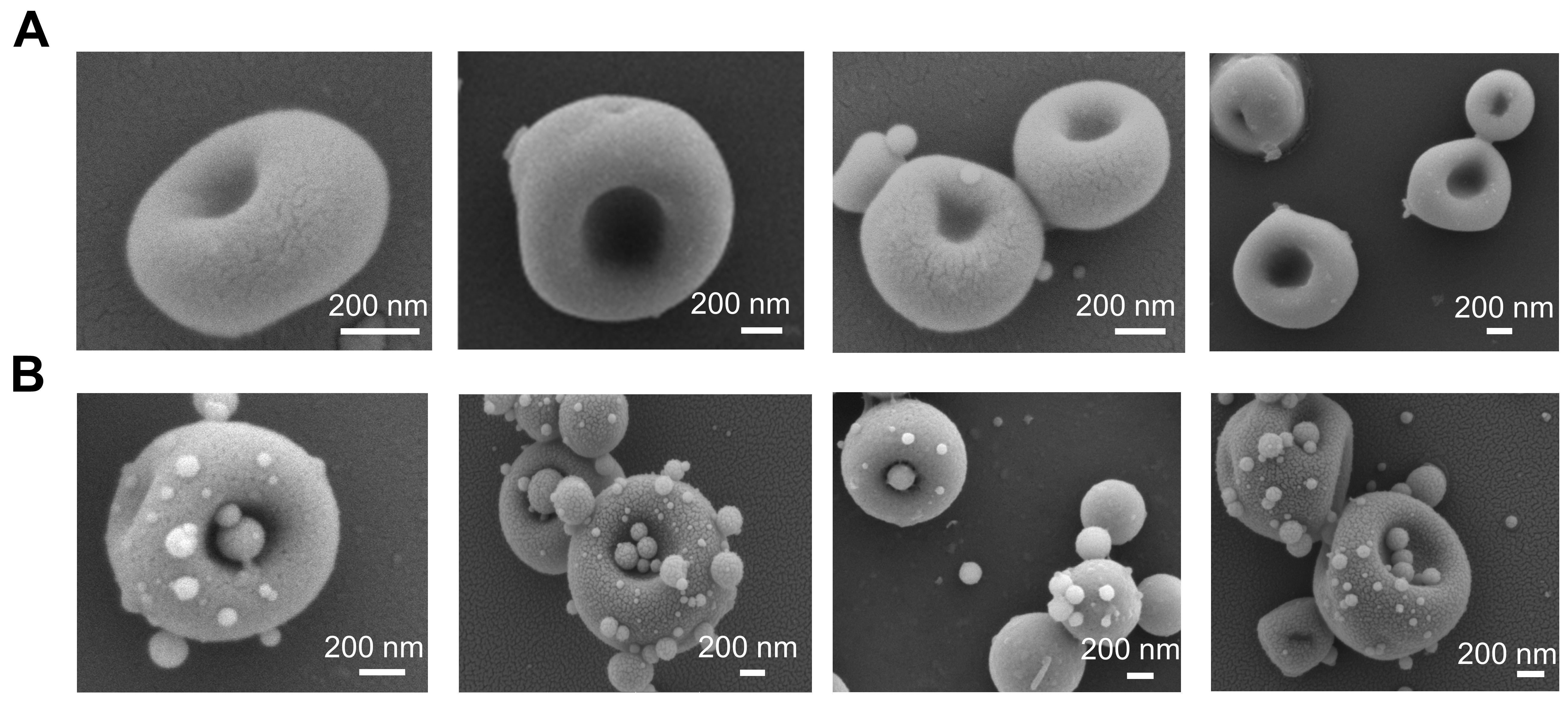


Figure S12. SEM images of 3D Hic-TiO_2_ A) and sEVs captured on BAIP modified Hic-TiO_2_ B).


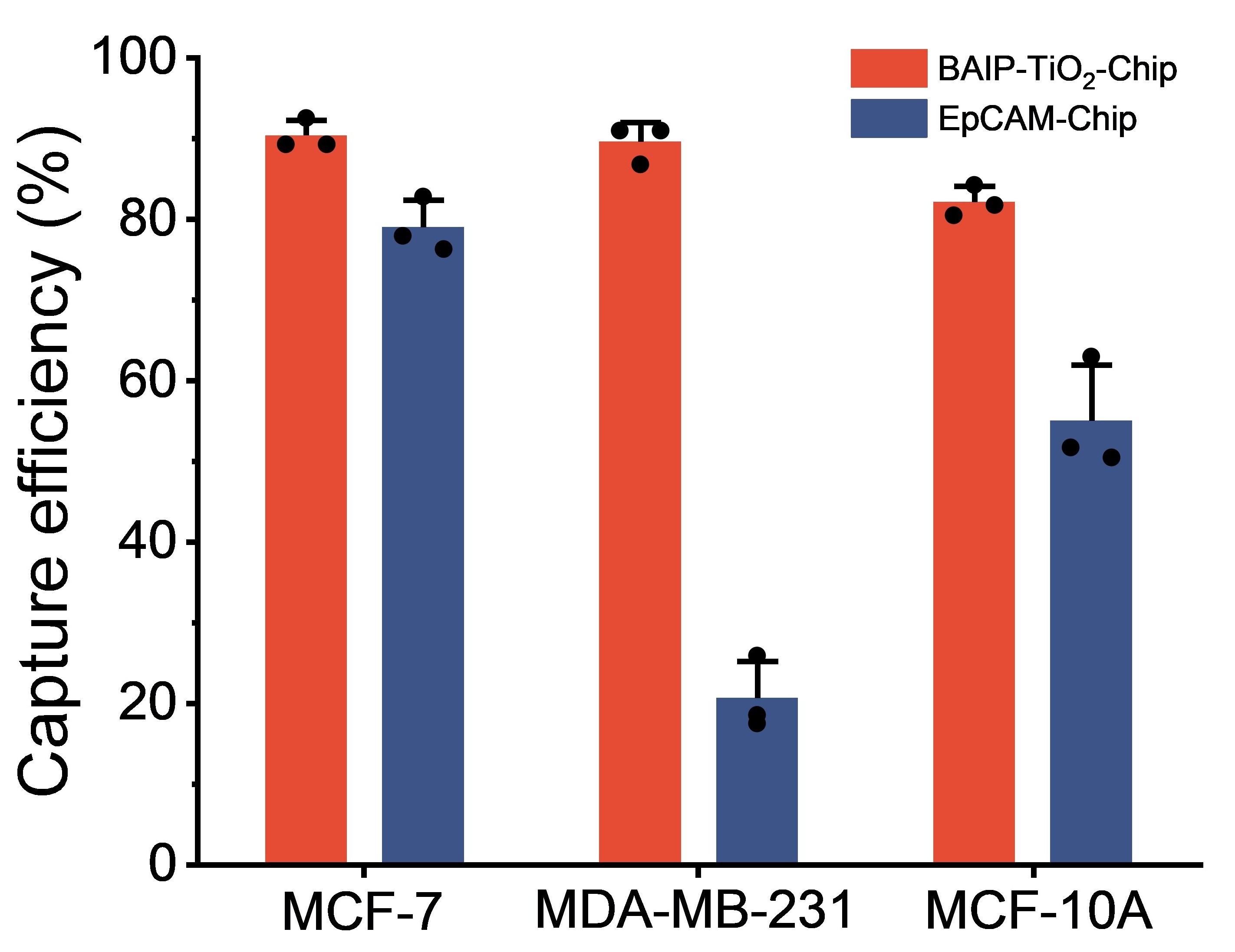


Figure S13. A comparative analysis of the capture efficiencies between the BAIP-TiO_2_-Chip and the EpCAM antibody functionalized TiO_2_-Chip was conducted using sEVs derived from MCF-7, MDA-MB-231, and MCF-10A cells.


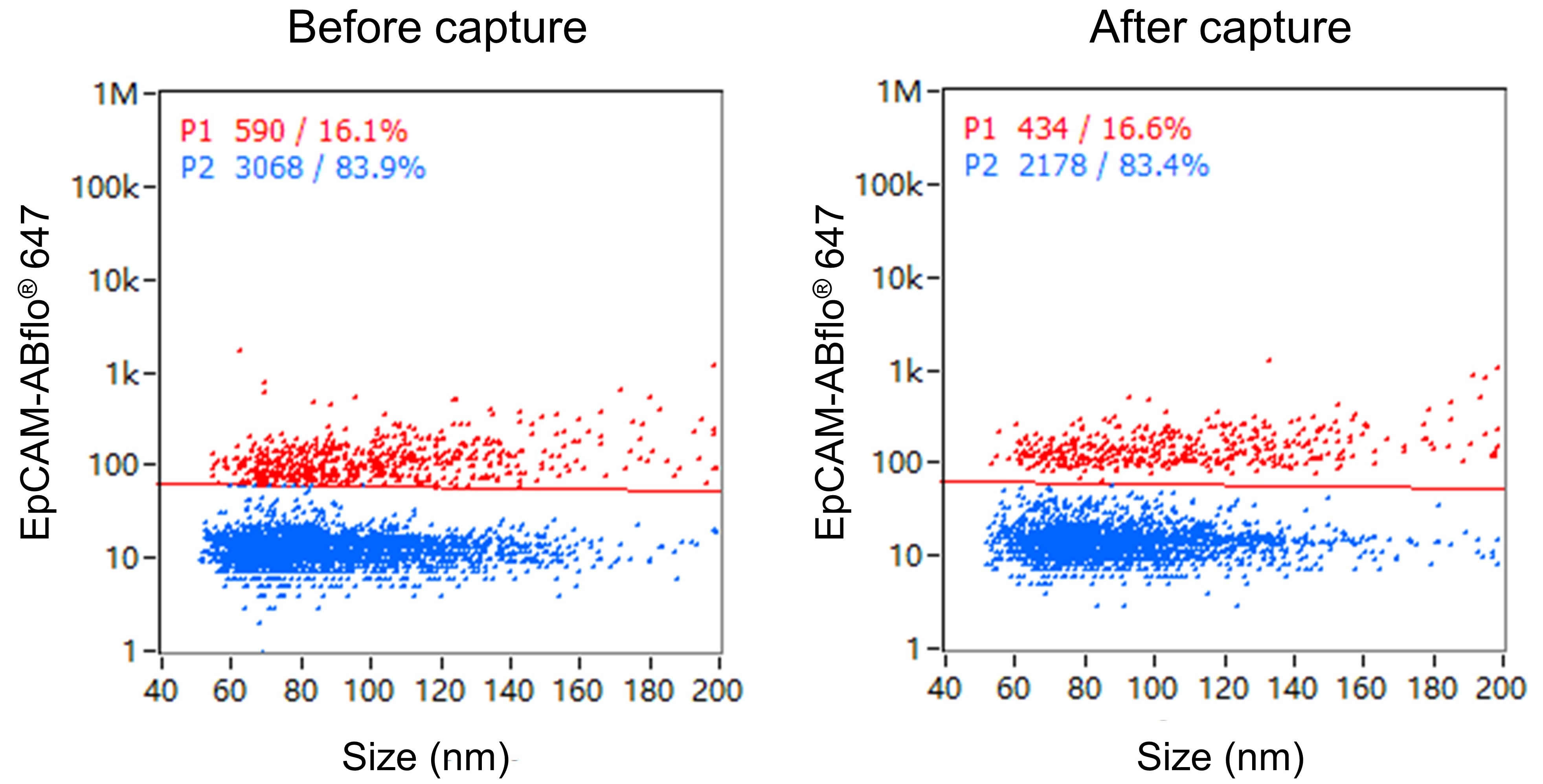


**Figure S14.** Using MCF-7-derived sEVs as a model, the proportion of EpCAM-positive sEVs in the total sEV population was evaluated before and after capture. The sEVs were labeled with a fluorescently conjugated anti-EpCAM monoclonal antibody (A22486, ABflo^®^ 647 Rabbit anti-Human CD326/EpCAM), followed by ultrafiltration purification and analysis by NanoFCM. The red area represented EpCAM-positive sEVs.


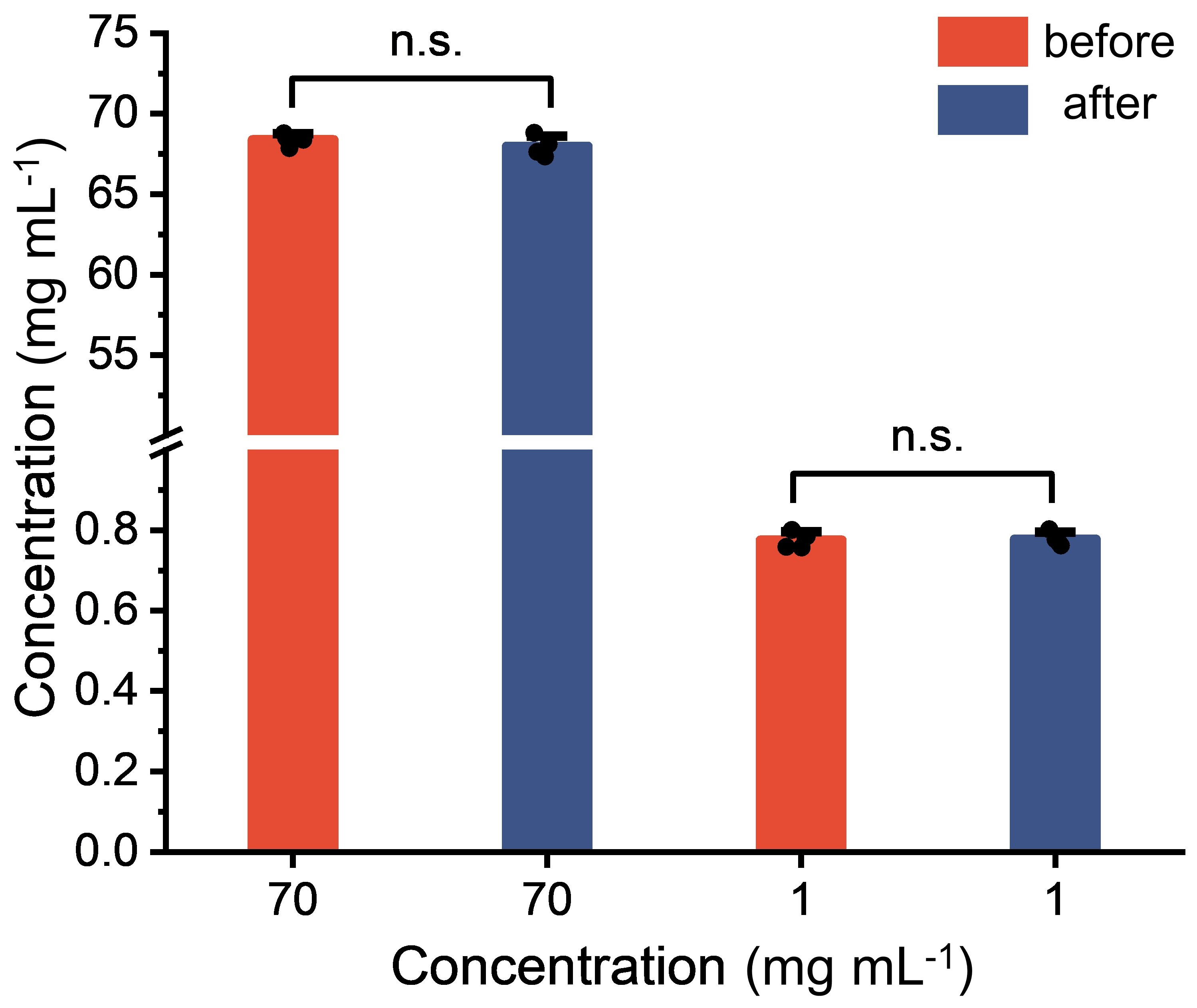


**Figure S15.** Evaluation of nonspecific albumin adsorption on the BAIP-TiO_2_-Chip. The chip was incubated with 50 μL of BSA at 70 mg mL^-1^ or 1 mg mL^-1^ at room temperature for 5 min. Residual BSA concentrations showed no significant change after incubation, indicating negligible adsorption. The x-axis “Concentration” represents the concentration of initial BSA without incubation with BAIP-TiO_2_-Chip, while the y-axis “Concentration” represents the concentration of the remained BSA after incubation with BAIP-TiO_2_-Chip. Data were represented as mean ± S.D. (n = 4). Statistical analysis was assessed by two-tailed Student’s t-tests; n.s. no significance.


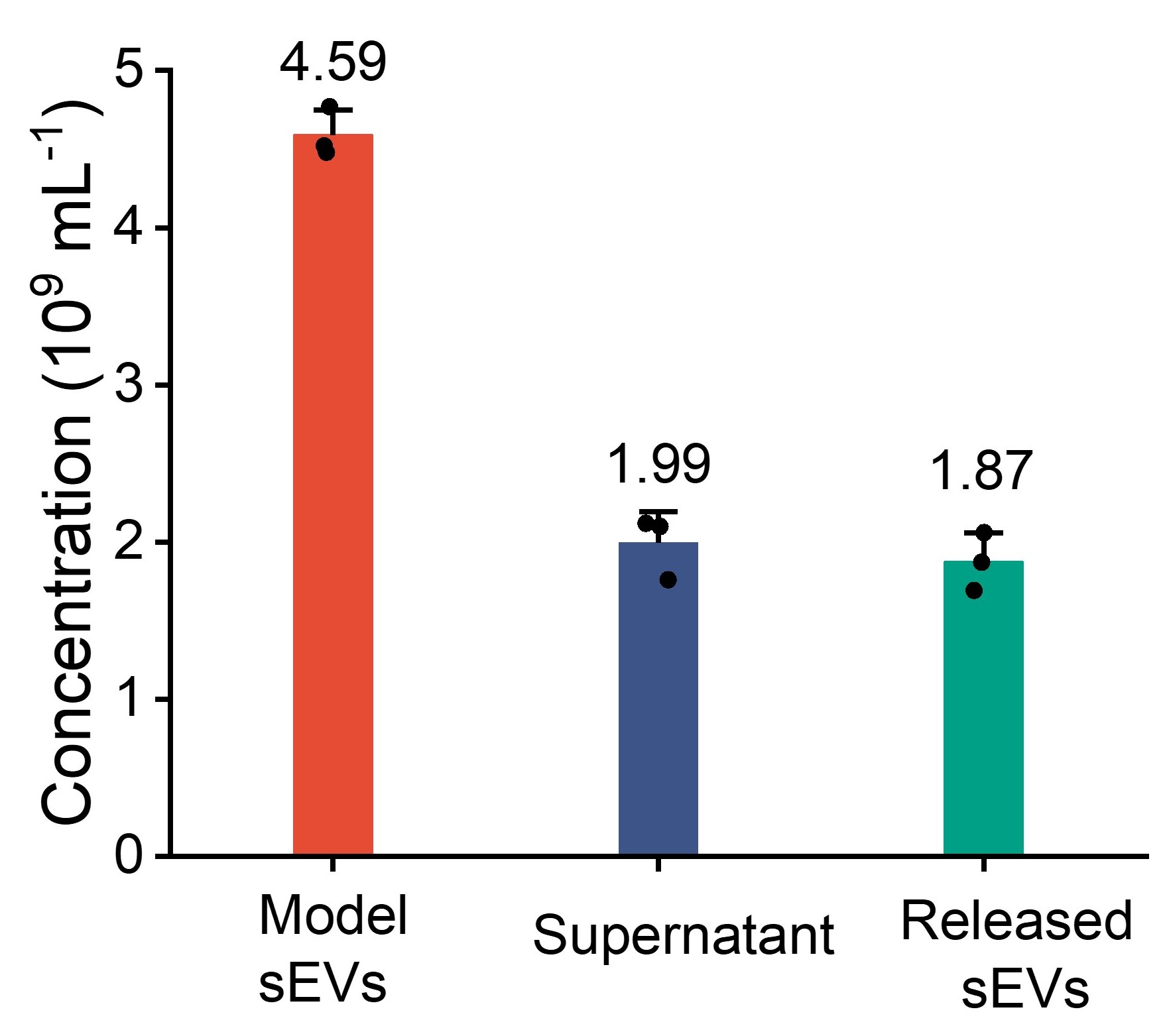


Figure S16. The concentrations of the initial sEVs, the residual sEVs and the released sEVs quantified using NanoFCM (10-fold dilution). The released efficiency was calculated to be 72.19% ± 7.09% according to Equation (3).


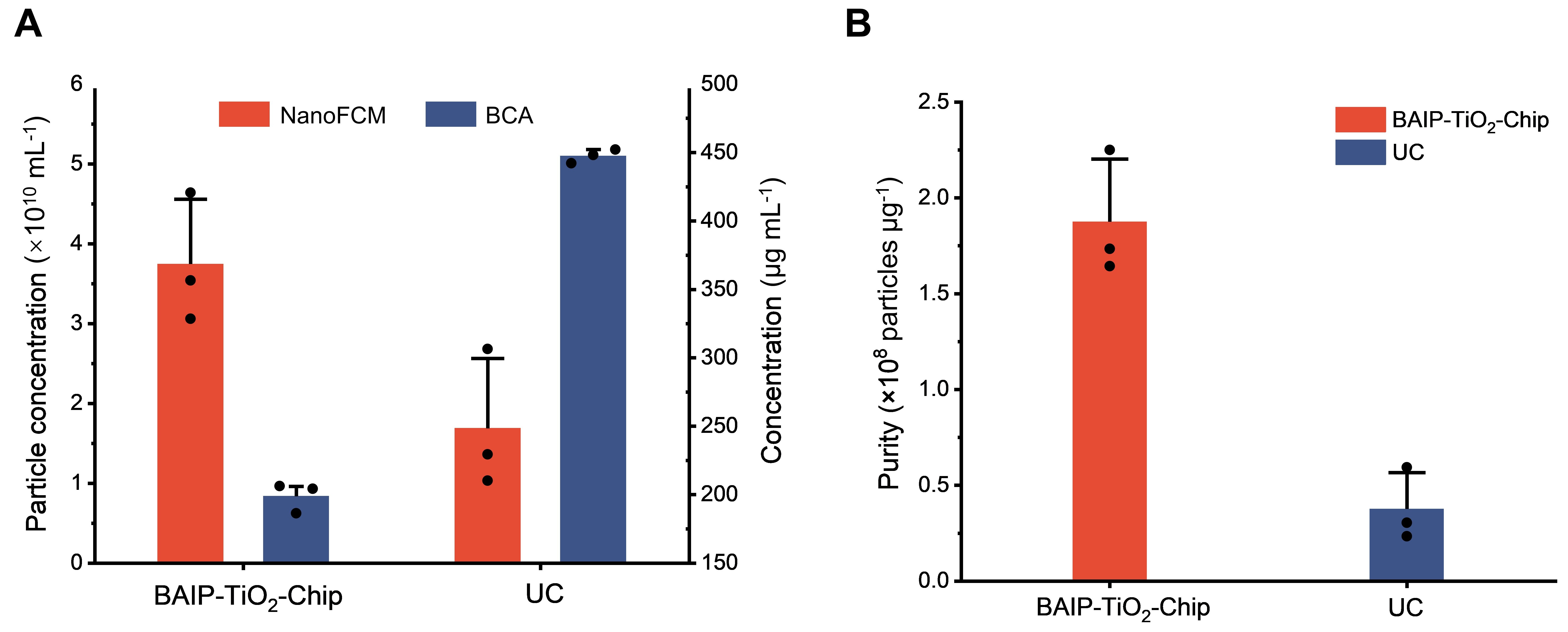


Figure S17. Evaluation of the sEVs purity. A) NanoFCM quantification of particle concentration (left bar chart) and protein concentration (right bar chart) in sEVs samples isolated from mimetic samples using the BAIP-TiO_2_-Chip and UC. B) Particle-to-protein ratios of sEVs obtained by the two methods.


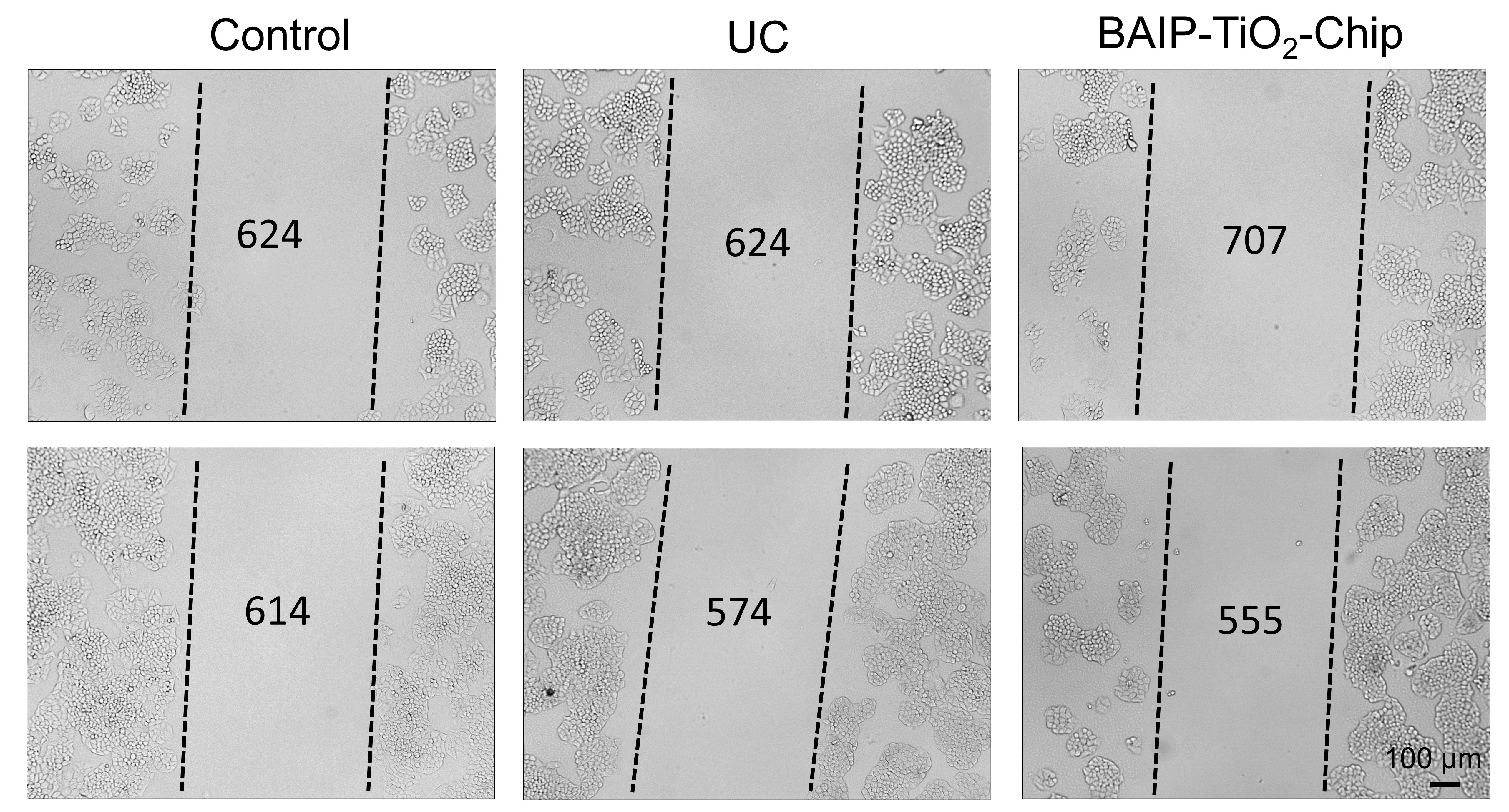


Figure S18. Migration was examined at 24 h after wounding. The absence of MDA-MB-231 sEVs was set as control. The experiments were performed with the same concentration of sEVs **prior to** isolation using UC and BAIP-TiO_2_-Chip.


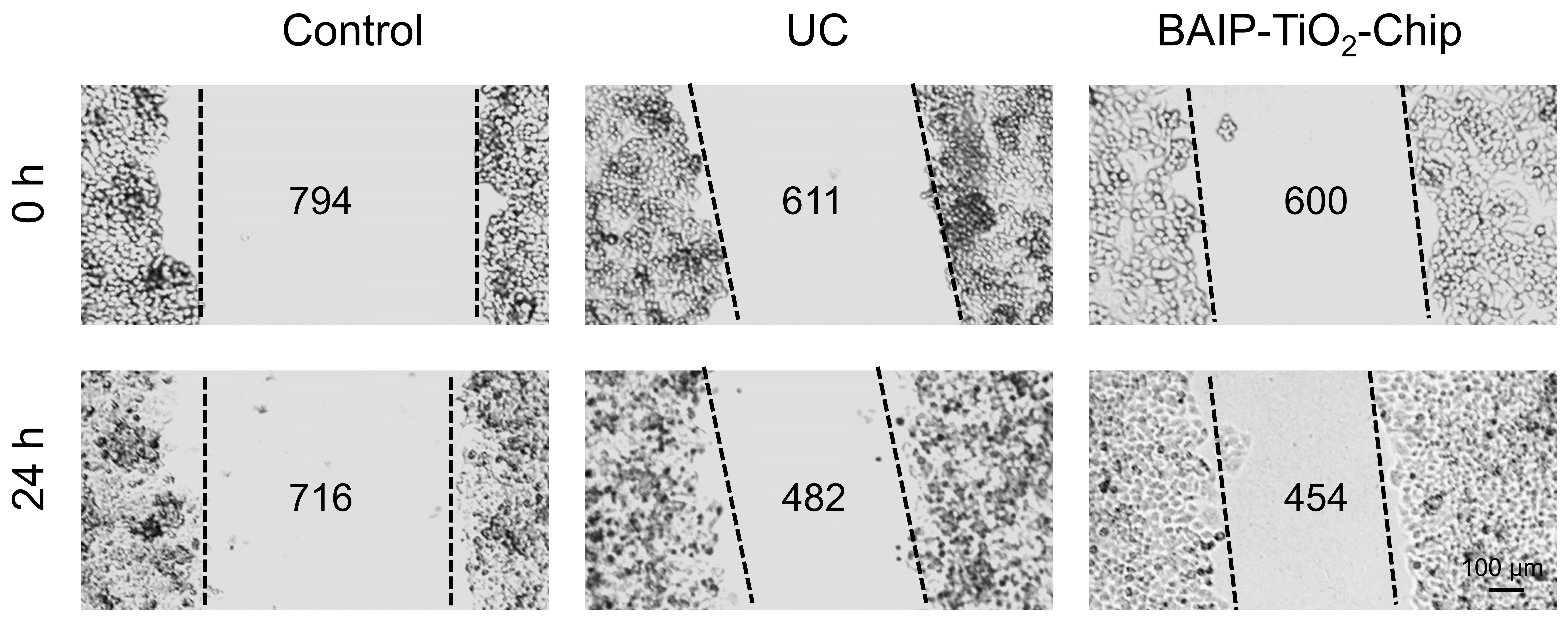


Figure S19. Migration was examined at 24 h after wounding. The absence of MDA-MB-231 sEVs was set as control. The experiments were performed with the concentration of sEVs adjusted to be consistent after isolation by UC and BAIP-TiO_2_-Chip.


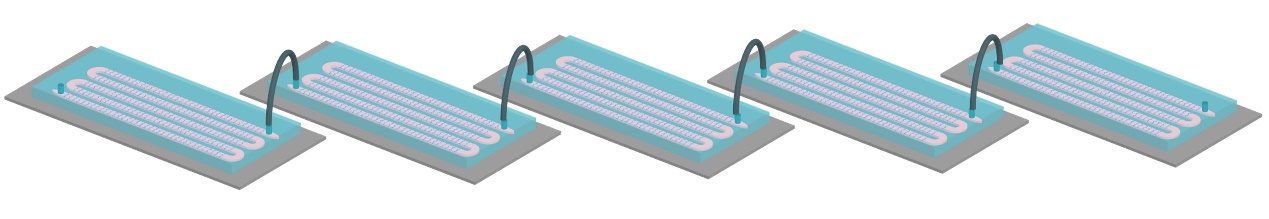


Figure S20. Schematic diagram of five BAIP-TiO_2_-Chips linked in series.

**
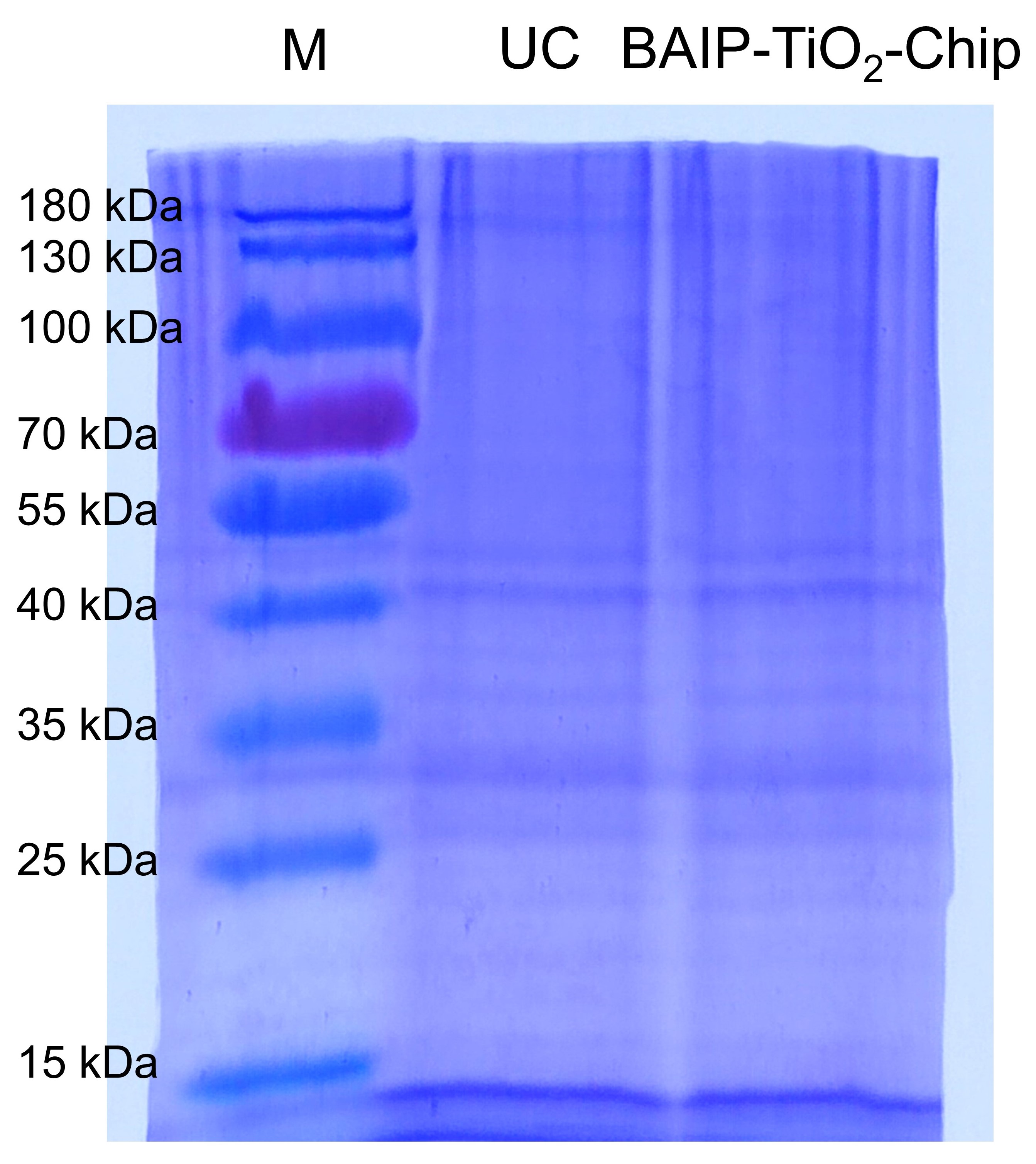
**

Figure S21. SDS-PAGE of proteins from model sEVs derived from MCF-7 cells isolated by UC and BAIP-TiO_2_-Chip.

**
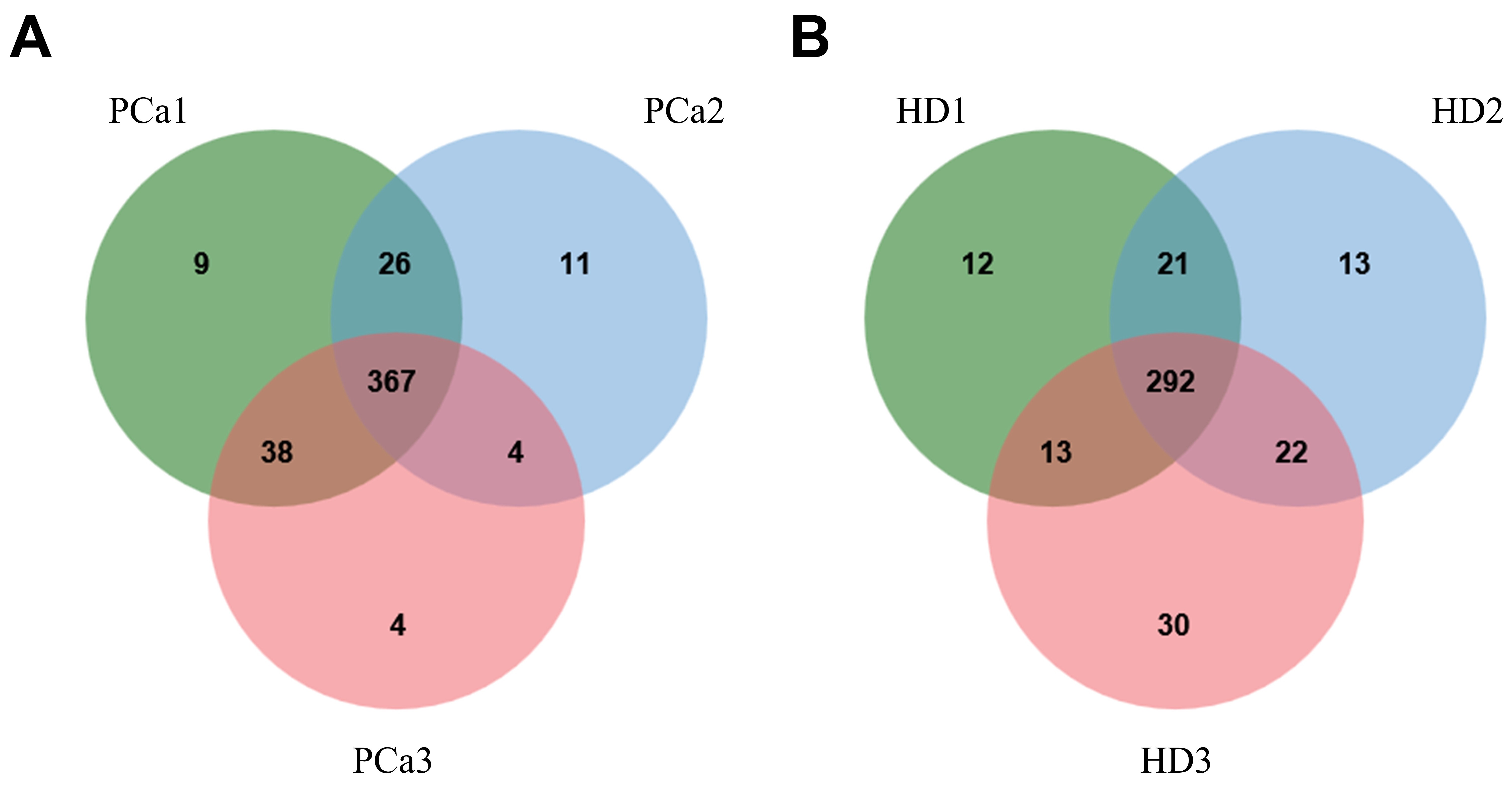
**

Figure S22. Venn diagram of the EV proteins detected in the three different HD groups A) and three different PCa groups B).

**
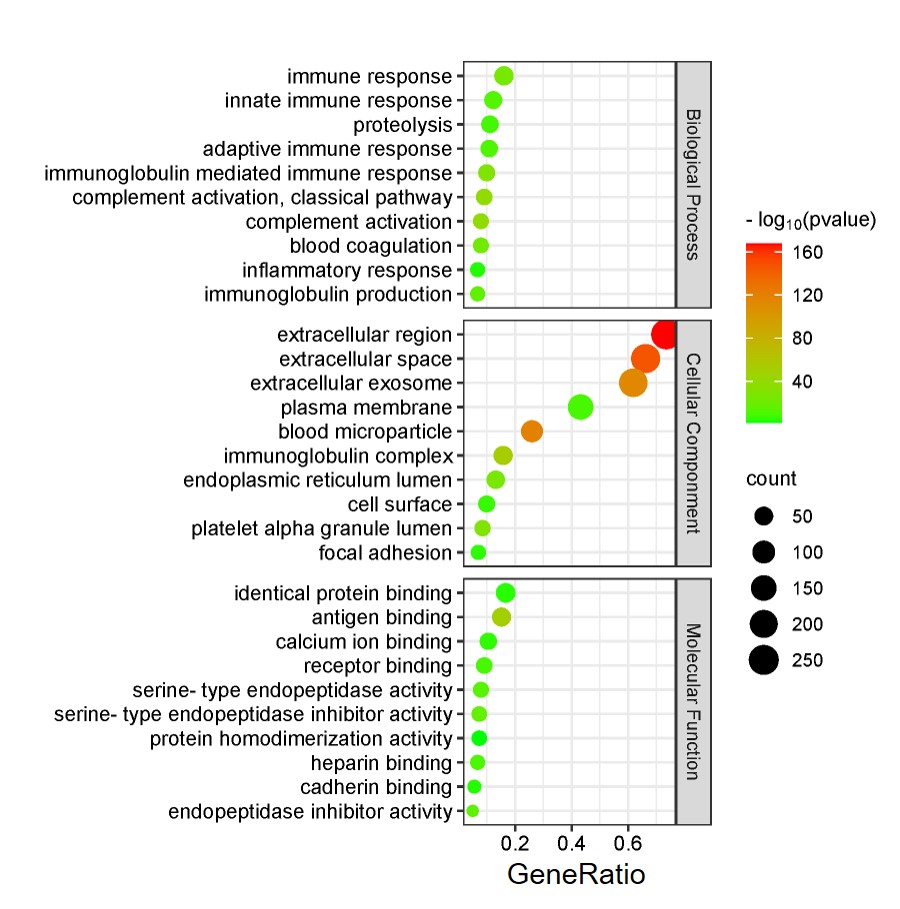
**

Figure S23. GO enrichment analysis of biological process, cellular component, and molecular function on total proteins of HD samples detected from more than half of the total samples.

**
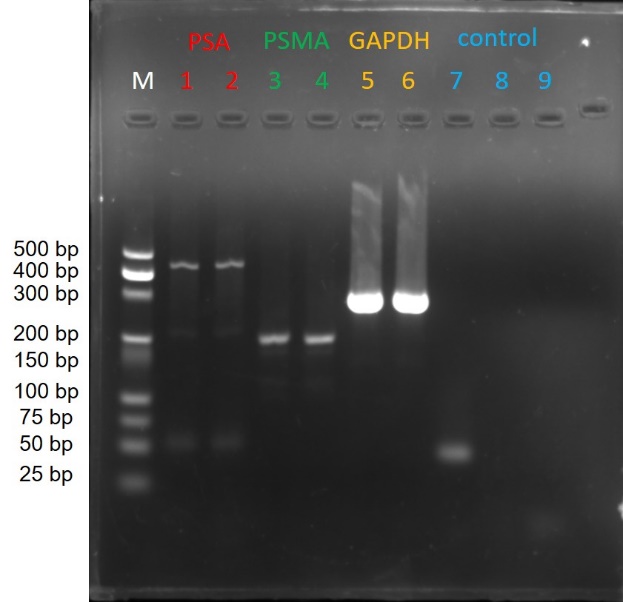
**

Figure S24. Agarose gel electrophoresis of the fragments from RT-PCR amplification of *PSA* mRNA (sEVs isolated by UC (line 1) and BAIP-TiO_2_-Chip (line 2)), *PSMA* mRNA (sEVs isolated by UC (line 3) and BAIP-TiO_2_-Chip (line 4)) and *GAPDH* mRNA (sEVs isolated by UC (line 1) and BAIP-TiO_2_-Chip (line 2)). Line 7-9 were RT-PCR amplification without cDNA adding but with *PSA* primer (line 7), *PSMA* primer (line 8) and *GAPDH* primer adding (line 9).


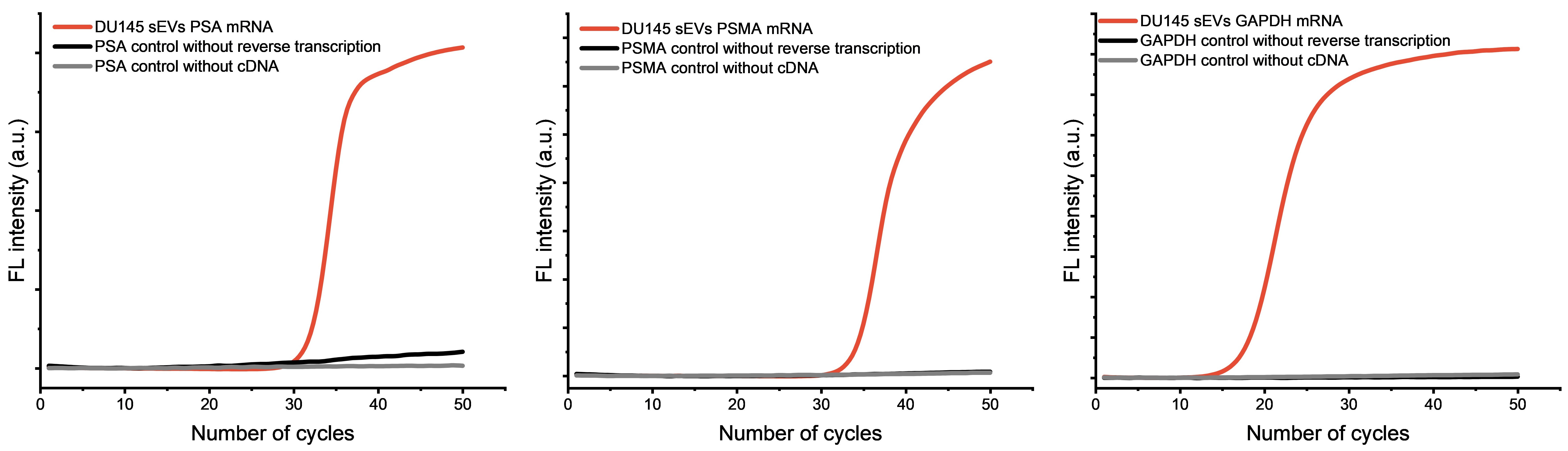


Figure S25. The qPCR detection of *PSA*, *PSMA*, and *GAPDH* mRNA in DU145-derived sEVs isolated by BAIP-TiO_2_-Chips, including no-template and no-RT controls.


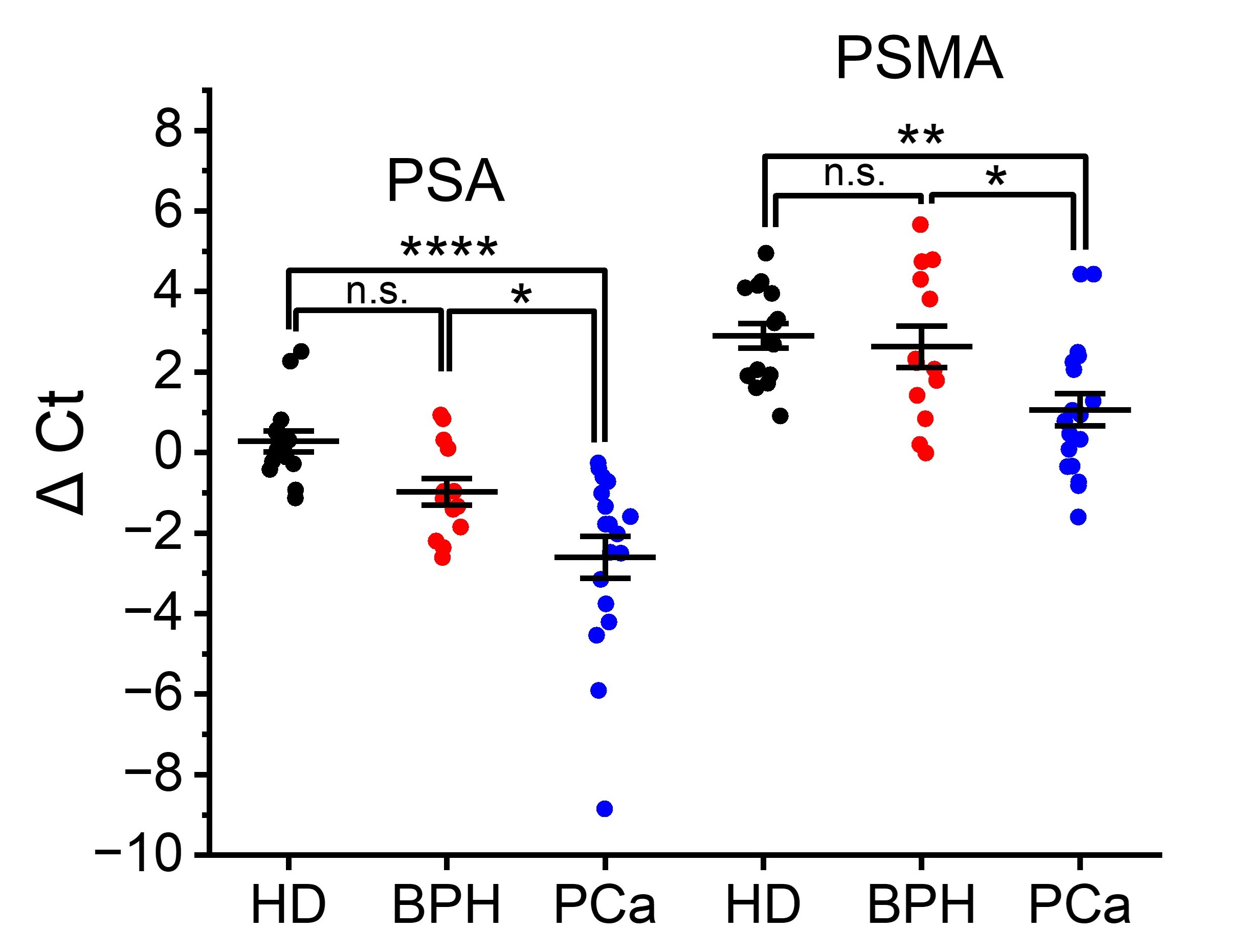


Figure S26. Significant elevation in sEVs *PSA* and *PSMA* mRNA level for PCa compared with BPH and HD.


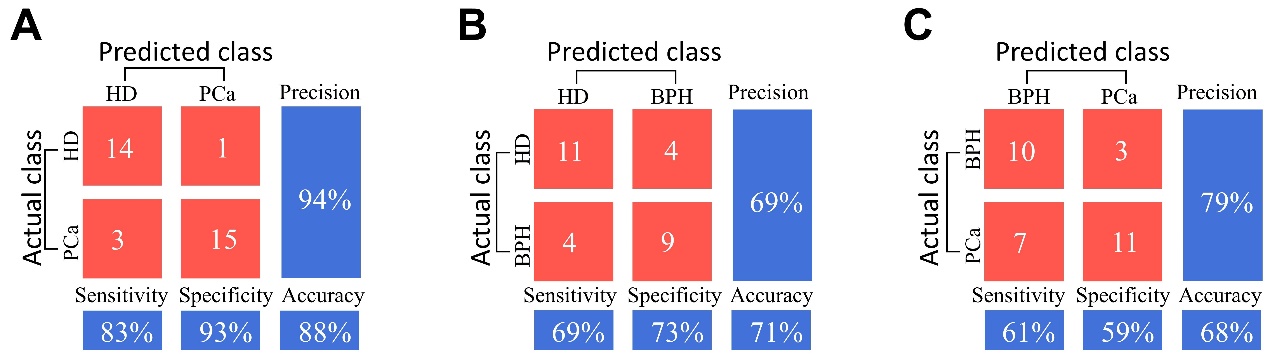


Figure S27. The LDA confusion matrices of EV-derived ΔCt*_PSA_* and ΔCt*_PSMA_* mRNA in HD and PCa A), HD and BPH B), and BPH and PCa C).

Table S1. The total proteins and EV proteins quantified by LC-MS/MS from the sEVs captured by BAIP-TiO_2_-Chip from clinical plasma samples.

| Sample No. | Total protein | EV protein | Proprotion of EV protein (%) |
| --- | --- | --- | --- |
| HD1 | 338 | 205 | 60.65 |
| HD2 | 348 | 214 | 61.49 |
| HD3 | 357 | 212 | 59.38 |
| Mean | 347 | 210 | 60.51 |
| PCa1 | 440 | 262 | 59.54 |
| PCa2 | 408 | 238 | 58.33 |
| PCa3 | 413 | 252 | 61.02 |
| Mean | 420 | 250 | 59.63 |

Table S2. Summary of HD, BPH and PCa patients.

| Sample | Age | PSA (ng mL^-1^) | PSA-GAPDH | PSMA-GAPDH |
| --- | --- | --- | --- | --- |
| HD1 | 70 | / | 0.07 | 3.31 |
| HD2 | 65 | / | 0.06 | 4.09 |
| HD3 | 61 | / | 0.16 | 1.93 |
| HD4 | 43 | / | 2.51 | 4.25 |
| HD5 | 53 | / | -0.22 | 1.72 |
| HD6 | 57 | / | 2.27 | 0.91 |
| HD7 | 80 | / | -0.11 | 1.91 |
| HD8 | 43 | / | 0.51 | 2.73 |
| HD9 | 41 | / | 0.31 | 4.95 |
| HD10 | 66 | / | -1.13 | 1.61 |
| HD11 | 55 | / | 0.57 | 3.22 |
| HD12 | 51 | / | -0.42 | 3.95 |
| HD13 | 66 | / | -0.28 | 4.15 |
| HD14 | 51 | / | -0.93 | 2.69 |
| HD15 | 51 | / | 0.81 | 2.06 |
| BPH1 | 70 | 8.76 | -1.41 | 5.66 |
| BPH2 | 69 | 18.702 | -2.61 | -0.01 |
| BPH3 | 73 | 22.351 | -1.15 | 2.23 |
| BPH4 | 67 | >1000 | 0.1 | 4.79 |
| BPH5 | 54 | 36.75 | -0.96 | 3.81 |
| BPH6 | 54 | 13.641 | -1.85 | 2.32 |
| BPH7 | 62 | 18.909 | 0.31 | 0.84 |
| BPH8 | 80 | 20.83 | -2.2 | 1.42 |
| BPH9 | 66 | 21 | 0.84 | 4.74 |
| BPH10 | 72 | 8.177 | 0.93 | 4.3 |
| BPH11 | 64 | 7.334 | -1.34 | 1.79 |
| BPH12 | 63 | 50.2 | -2.36 | 0.2 |
| BPH13 | 59 | / | -0.97 | 2.07 |
| PCa1 | 76 | 821.35 | -5.91 | -0.82 |
| PCa2 | 65 | 7.933 | -8.85 | 4.43 |
| PCa3 | 68 | 12.326 | -4.54 | 0.08 |
| PCa4 | 73 | / | -1.78 | 2.4 |
| PCa5 | 63 | 72.856 | -1.78 | -0.35 |
| PCa6 | 54 | 4.68 | -0.61 | 0.94 |
| PCa7 | 57 | 5.213 | -2.5 | 4.43 |
| PCa8 | 58 | 8.585 | -2.02 | 0.46 |
| PCa9 | 58 | 8.84 | -3.76 | -1.6 |
| PCa10 | 51 | / | -4.21 | 2.25 |
| PCa11 | 74 | 215.73 | -0.39 | 0.33 |
| PCa12 | 69 | 18.05 | -0.26 | 1.05 |
| PCa13 | 54 | 8.586 | -1.34 | 2.06 |
| PCa14 | 76 | 31.412 | -2.48 | -0.34 |
| PCa15 | 65 | 65.913 | -3.15 | -0.73 |
| PCa16 | 74 | >100 | -1.59 | 2.49 |
| PCa17 | 61 | 12.5 | -1.01 | 1.28 |
| PCa18 | 65 | / | -0.72 | 0.78 |

Table S3. The comparison of BAIP-TiO_2_-Chip and traditional methods for separation of sEVs.

| Techniques | Isolation efficiency | Purity | Time | Sample volume | Ref |
| --- | --- | --- | --- | --- | --- |
| Ultrafiltration | 10%-80% | Low | 0.5-1 h | μL-mL | [1,2] |
| Polymer-based precipitation separation | ~60%-90% | Low | 12-16 h | μL-mL | [3-5] |
| BAIP-PS | ~76% | High | 20 min | μL | [6] |
| Ultracentrifugation | 15% | High | ~4 h | mL | This study |
| BAIP-TiO_2_-Chip | >90% | High | 5 min | μL | This study |

Table S4. Primers information of RT-qPCR.

| Gene | Forward primer (5’-3’) | Reverse primer (5’-3’) |
| --- | --- | --- |
| *PSA* | GCGTGATCTTGCTGGGTCGG | AGAATCACCCGAGCAGGTGCT |
| *PSMA* | AGGGGCCAAAGGAGTCATTC | CTCTGCAATTCCACGCCTAT |
| *GAPDH* | CGTCTTCACCACCATGGAGA | CGGCCATCACGCCACAGTTT |

**References**

[1] L. Balaj, N. A. Atai, W. Chen, et al., “Heparin Affinity Purification of Extracellular Vesicles,” *Scientific Reports*, 5 (2015): 10266, https://doi.org/10.1038/srep10266.

[2] R. J. Lobb, M. Becker, S. W. Wen, et al., “Optimized Exosome Isolation Protocol for Cell Culture Supernatant and Human Plasma,” *Journal of Extracellular Vesicles* 4 (2015): 27031, https://doi.org/10.3402/jev.v4.27031.

[3] T. Soares Martins, J. Catita, I. Martins Rosa, et al., “Exosome Isolation from Distinct Biofluids Using Precipitation and Column-Based Approaches,” *PloS One* 13 (2018): e0198820, https://doi.org/10.1371/journal.pone.0198820.

[4] M. Grunt, A. V. Failla, I. Stevic, et al., “A novel assay for exosomal and cell-free miRNA isolation and quantification,” *RNA Biology* 17 (2020): 425, https://doi.org/10.1080/15476286.2020.1721204.

[5] M. Chang, Y. J. Chang, P. Y. Chao, et al., “Exosome Purification Based on PEG-Coated Fe_3_O_4_ Nanoparticles,” *PloS One* 13 (2018): e0199438, https://doi.org/10.1371/journal.pone.0199438.

[6] L. Wang, Z. Gong, M. Wang, et al., “Rapid and unbiased enrichment of extracellular vesicles via a meticulously engineered peptide,” *Bioactive Materials* 43 (2025): 292, https://doi.org/10.1016/j.bioactmat.2024.09.023.
